# OmpA Specifically Modulates the Activity of Enzymes that Reside in the Crowded Bacterial Outer Membrane

**DOI:** 10.1016/j.jmb.2025.169346

**Published:** 2025-07-14

**Authors:** Jonathan M. Machin, Neil A. Ranson, Sheena E. Radford

**Affiliations:** **Astbury Centre for Structural Molecular Biology**, School of Molecular and Cellular Biology, Faculty of Biological Sciences, University of Leeds, Leeds LS2 9JT, UK

**Keywords:** outer membrane protein (OMP), OMP–OMP interactions, OMP islands, bacterial outer membrane

## Abstract

The outer membrane (OM) of lipopolysaccharide (LPS) containing-diderm bacteria is crowded with outer membrane proteins (OMPs) that reside in a membrane that is relatively rich in protein and poor in lipid. As a consequence, extensive interactions between OMPs occur. Yet, how these interactions affect OMP function remains unexplored. Here, we examine the effect of OmpA on the activity of three different OMP enzymes, OmpLA (a phospholipase), PagP (a palmitoyltransferase) and OmpT (a protease). We show that OmpA-OmpT interactions enhance the activity of OmpT, and that this catalytic enhancement is mediated via their extracellular loops, an effect that is not observed with other common OMPs, including OmpF and OmpX. In contrast, OmpA specifically reduces the activity of PagP, while OmpLA activity shows no significant change. Possible interactions between the abundant *E. coli* OMPs (OmpA, OmpF/C, OmpT, OmpX, MipA) and all other *E. coli* OMPs were screened via Alphafold predictions, with the results suggesting that smaller OMPs are generally more promiscuous interactors, and identifying new interactions that may plausibly form in the OMP-rich islands in the OM. Together, the results identify a previously underappreciated role for specific OMP–OMP interactions in modulating protein function in the OM, and highlight how evolution may have exploited the high local concentrations of abundant OMPs in the OM to tune enzyme activity.

## Introduction

Lipid and protein constituents of biological membranes are typically constrained and organised by additional levels of higher-order membrane structure [1,2]. In the outer membrane (OM) of lipopolysaccharide-containing (LPS)-diderm bacteria this is particularly striking, with lipids and proteins partially phase-separating into outer membrane protein (OMP) islands which can be >500 nm in diameter [3], and LPS patches, typ ically ∼55 nm diameter [4], each significantly enriched in their own component [4]. A combination of the low lipid to protein ratio (LPR) of ∼8:1 in the OM (c.f. ∼32:1 for the LPS-diderm inner membrane [5]), the stable arrays formed by trimeric porins [6], the relative immobility of the large LPS layer and the LPS oligosaccharides which cluster together via divalent cation-mediated interactions [7], means that very limited diffusion is possible [8]. Indeed, in general, the rate of OMP diffusion scales with the local membrane protein density, although specific proteins can either be tightly trapped or more free to diffuse [9]. OMP–OMP interactions are thought to be mediated by LPS [10], but given the scarcity of lipids and their partition into lipid-rich domains, many direct protein-protein interactions must occur in OMP islands, for example as characterised for OmpF-BtuB [11]. The rapid membrane insertion of OMPs (about two thirds of which are either OmpA or the trimeric porins OmpF/OmpC), which typically take only a few seconds to partition into the membrane via the BAM complex [12] and occurs in ‘folding precincts’ consisting of multiple copies of BAM [13], further drives the formation of OMP–OMP interactions in the OM. This tight packing of proteins and the many interaction points between individual LPS moieties and multiple OMPs helps to generate the primary barrier function of the OM. Together, the OM emerges as a mosaic of OMP and lipid islands with protein-rich domains characterised by protein-protein interactions involving the highly abundant OmpA, OmpF and OmpC, with other OMPs which play diverse functional roles (e.g. enzymatic) beyond scaffolding and porins.

**Figure 1:**
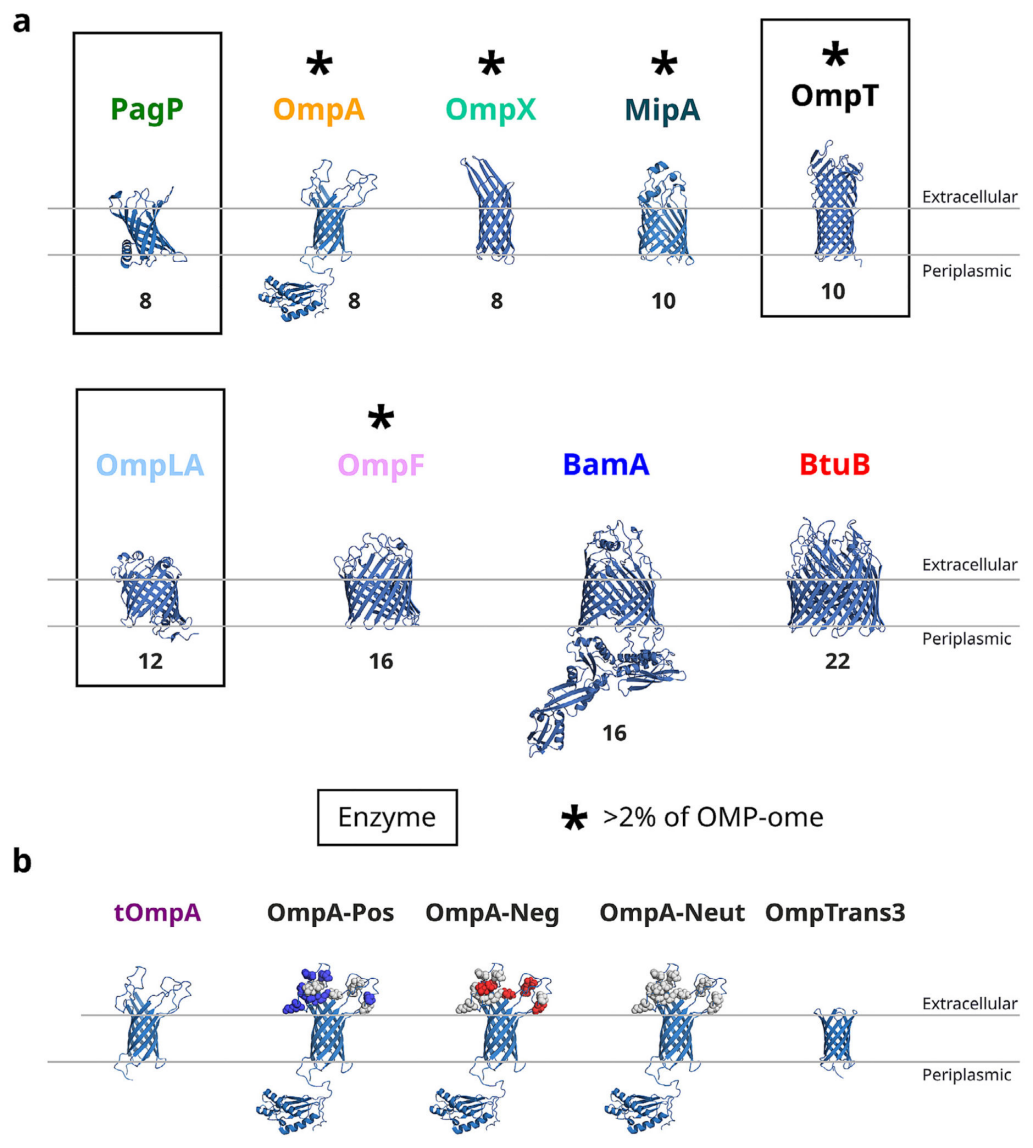
OMP and OmpA variants used in this study. **(a)** *E. coli* WT OMPs used in this study, the three OMP enzymes are boxed and * indicates OMPs typically greater than 2% of the OMPome. Barrel strand number is indicated beneath each structure. (PDBs: PagP: 3GP6, OmpA: 1G90/2MQE, tOmpA: 1G90, OmpX: 1QJ8, OmpT: 1I78, OmpLA: 1QD5, OmpF: 1MPF, BamA: 5D0O, BtuB: 1NQE, MipA was generated by Alphafold). **(b)** OmpA variants used in this study. tOmpA lacks the C-terminal domain, OmpA-Pos, OmpA-Neg and OmpA-Neut have charge altering mutations in the extracellular loops (Asp/Glu are mutated to neutral residues in OmpA-Pos, Lys/Arg are mutated to neutral residues in OmpA-Neg and all charged residues are mutated to neutral residues in OmpA-Neut (see Methods, mutated residues shown as white spheres, charges as blue (positive) or red (negative) spheres). OmpTrans3 has short extracellular turns instead of the natural longer loops [38]. All structures shown on the same scale.

There are three OMP enzymes in K12 *E. coli*[14]. All are constitutively expressed and implicated in virulence [15–17], and all play a key role in the rapid management of cellular threats before adaptive responses can adjust the OM composition [14]. The protease OmpT is a relatively abundant (∼6% of the OMPome [18]) 10-stranded OMP (Figure 1 a) responsible for cleaving host antimicrobial peptides [19]. The activity of OmpT is substantially enhanced (>10×) via interaction with LPS [20]. The phospholipase OmpLA (or PldA, typically ∼0.2% of the OMPome [18]) is a 12-stranded barrel (Figure 1a) that degrades phospholipids that are erroneously found in the outer leaflet of the OM, generating their fatty acid and lysophospholipid constituents [21,22]. OmpLA functions as a constitutive dimer that strictly requires divalent cations for dimerization and activ ity [23]. The third enzyme, PagP (typically <0.1% of the OMPome [18]), is an eight-stranded lipid A palmitoyltransferase (transferring a palmitoyl group from phospholipids to LPS) [24] (Figure 1a), an adaptation that stiffens the OM and, among other roles, helps protect against cationic antimicrobial peptides [16]. In the absence of acceptor substrate (i.e. LPS) PagP also displays a slow phospholipase activity [25,26]. Interestingly, although all three enzymes are implicated in pathogenesis in *E. coli*, one or more of these enzymes is genetically lost or inactivated in a range of other pathogens [27,28]. This suggests that their activity may be incompatible with some pathogenic states, and highlights the likely advantages to a bacterium for precise modulation of OM enzyme activity. However, while the effects of lipid interactions on the activity of all three of these enzymes have been studied [29–31], how interactions with other OMPs affects their function remained unexplored.

The OMP content of the OM of *E. coli* is dominated by just a few of the ∼65 OMPs known to be expressed in the bacterium, with only six OMPs (OmpF, OmpC, OmpA, OmpX, OmpT, MipA) estimated to be present at >3% of the total OMP content when grown in rich media. By contrast ∼70% of the total OMPs are formed from monomeric OmpA and the trimeric porins OmpF/OmpC regardless of environmental conditions [18]. Therefore, the majority of OMPs in the OM must contact OmpA and/or a trimeric porin (either directly or via lipid/LPS), presumably with long-lived interactions due to the highly constrained diffusion therein [8,9]. Furthermore, OmpA has recently been shown to be critical for the formation of the low-diffusion OMP lattice [32], presumably by mediating promiscuous OMP–OMP interactions. Given these properties, it is plausible that evolution may have exploited common interactions to facilitate and modulate specific OMP functions. Indeed, functionality beyond their canonical role has been described for some common OMPs. For example, OmpC interacts with the lipoprotein MlaA to support the Mla lipid transport pathway [33,34], and OmpA’s periplasmic C-terminal domain has been shown to interact with RcsF, helping regulate the Rcs stress response [35]. However, there remains no examples of functional intra-membrane ‘moonlighting’ interactions between OMPs, despite the apparent evolutionary opportunity this presents.

Here we explore transmembrane interactions among OMPs, focusing on the role of OmpA in modulating catalysis by the three OM enzymes from K12 *E. coli* (PagP/OmpLA/OmpT) using enzyme assays in proteoliposomes created to contain different concentrations of each OMP. We show that OmpA doubles the activity of OmpT by increasing substrate affinity in a specific, 1:1 interaction that is not observed with other common OMPs, and use structural modelling and mutational analysis to map the OmpA–OmpT binding site. By contrast, OmpA specifically reduces the activity of PagP, but has no effect on the activity of OmpLA. We then screened an array of >500 possible OMP–OMP interactions between the abundant OMPs of the *E. coli* OM and the general OMPome using Alphafold to find other high-confidence predicted OMP–OMP interactions in the OM. Together, these data highlight the importance of considering both specific and non-specific OMP–OMP interactions for OMP function, and hints at how evolution has exploited the near-infinite local concentrations of highly abundant proteins to modulate enzymatic function.

## Results

### DMPG facilitates the intrinsic folding of all OMPs

To study OMP–OMP interactions systematically *in vitro*, a membrane system that is competent to fold many OMPs at relatively low LPR (here ∼640:1 mol/mol) and high yield is required. While short chain phosphatidylcholine (PC) lipids have commonly been used for *in vitro* folding studies of OMPs [36], phosphatidylglycerol (PG)-containing lipids, in general, support higher folding yields, enhanced stability of the folded state and more rapid folding kinetics (important for minimising aggregation during folding [37]). Thus, the folding of a range of OMPs into 100 nm DMPG liposomes was assessed. An array of different *E. coli* OMPs was tested, including five of the six OMPs with protein counts estimated as >3% of all OMPs (OmpA, OmpF, OmpX, OmpT, MipA; the other highly abundant protein OmpC, is a homolog of OmpF), the other two enzymes in the *E. coli* OM (OmpLA and PagP), BamA (the core OMP of the BAM complex, required for the *in vivo* folding of all these OMPs), and BtuB (a 22-stranded OMP which is a TonB-dependent transporter), Figure 1a. The transmembrane region of OmpA (tOmpA), which is a well-studied model used in *in vitro* OMP folding assays was also included, along with three variants of OmpA in which the charge in its extracellular loops are altered, and one (OmpTrans3) in which the four extracellular loops are replaced with short turns [38], Figure 1b. These OMPs span a range of sizes (8–22 strands, 18–87 kDa) and functions (e.g. enzymatic activity, active and passive nutrient channels, adhesion factors, peptidoglycan interactions).

Measuring the folding kinetics of the nine OMPs shown in Figure 1a via tryptophan fluorescence shows successful folding for all, but one, of these proteins, with analysis of the spectra of the folded product and cold SDS-PAGE folded-unfolded band-shift assays confirming successful folding and insertion into the lipid bilayer at high efficiency (>90%) (Figure S1). (PagP did not fold under these conditions, consistent with previous results [39]). Although abundant (∼3%) in the native OM, MipA has not been previously characterised *in vitro*. SDS-PAGE indicated that it does not show a folded-unfolded band-shift in cold SDS PAGE (a common property usually observed for OMPs [37]), although an additional faint, presumably dimer, band appeared in the unboiled sample (Figure S2a). Nonetheless, far UV circular dichroism (CD) spectra of MipA refolded into dodecyl maltoside (DDM) detergent micelles or DMPG liposomes demonstrate a strong β-sheet signal compared to the unfolded protein in 8 M urea, consistent with β-barrel formation (Figure S2b). Together these data demonstrate the utility of DMPG liposomes (when in low concentrations of urea (e.g. 0.5 M) and at the gel-liquid transition temperature (23.5 °C)), to facilitate near-universal, high yield, folding for a wide range of OMPs of many sizes and complexities. Hence this lipid system was chosen to investigate the effect of protein-protein interactions in OMP function.

### The extracellular loops of OmpA enhance OmpT activity

**Figure 2:**
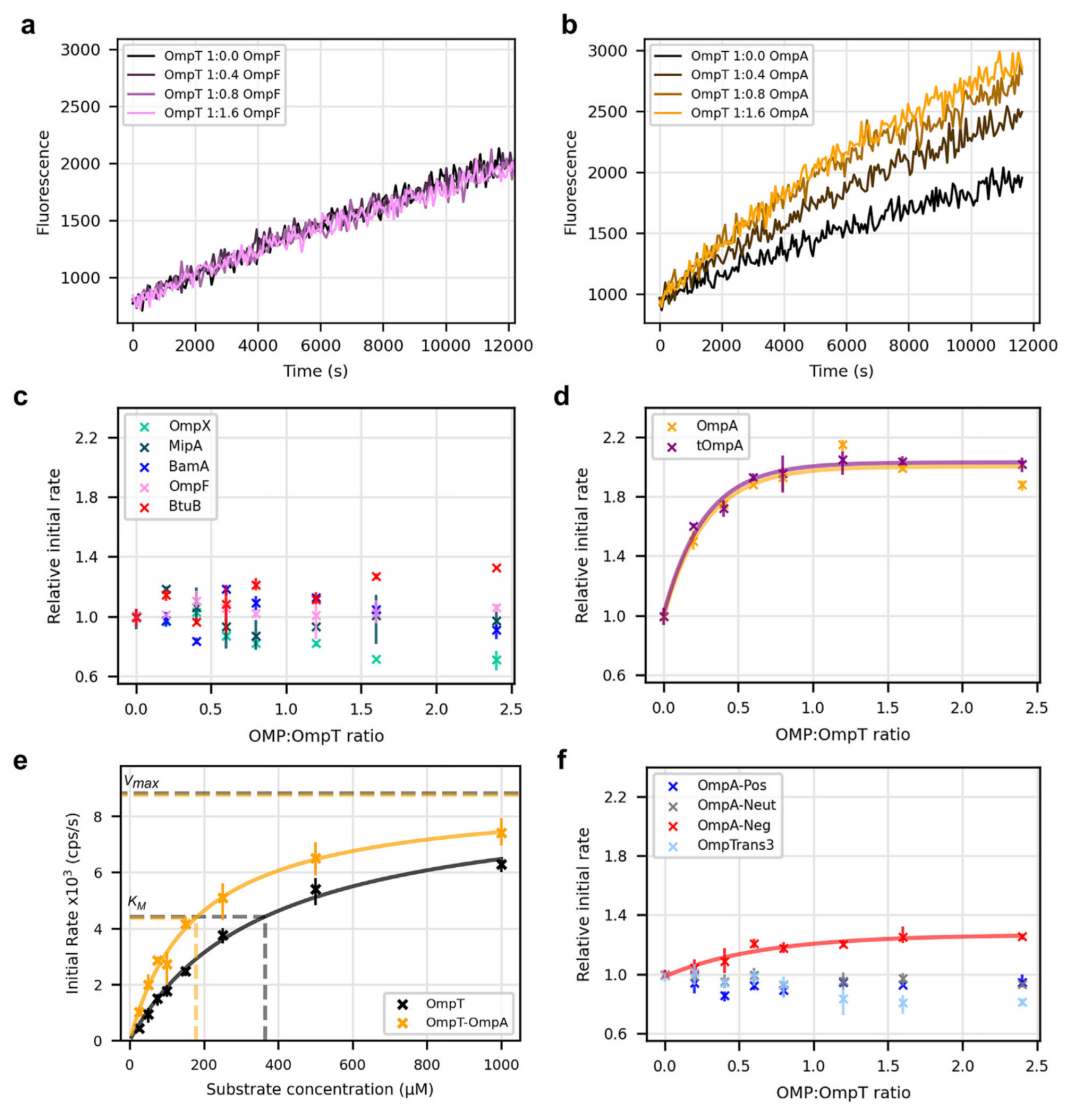
OmpA specifically enhances OmpT activity. OmpT activity traces in the presence of increasing amounts of **(a)** OmpF or **(b)** OmpA. Initial rate of OmpT activity in the presence of increasing concentrations of **(c)** OmpX, MipA, BamA, OmpF, or BtuB or **(d)** OmpA or tOmpA, **(c)** and **(d)** are plotted on the same scale to aid comparison. (**a-d** with 50 μM peptide substrate, 0.05 μM OmpT). **(e)** Substrate titration of the initial rate of OmpT activity in the presence or absence of OmpA. Fitting Michaelis-Menton kinetics allows derivation of *K*_M_ (lower left dashed lines) and V_max_ (upper dashed lines). OmpA 0.1 _|_ M, OmpT 0.05 _|_ M) (Methods). **(f)** Initial rate of OmpT activity in the presence of increasing concentrations of OmpA-Pos, OmpA-Neg, OmpA-Neut or OmpTrans3 (0.05 μM OmpT, 50 μM substrate). The data for OmpA-Neg are fitted to a single-phase exponential (red line). (*n* ≥ 3 in all experiments, error bars are data range).

The OmpT protease is the most highly abundant of the OMP enzymes in *E. coli* [18]. To explore the possible role of intermolecular OMP interactions in altering its activity, OmpT was folded into proteoli-posomes of defined LPR (6000:1, ∼20 OmpT molecules per liposome), in the presence of a 0-2.4-times molar excess of a second OMP (Figure 2a, b). Determining the initial rate of OmpT activity via cleavage of a fluorescent peptide substrate (Methods) [40] showed that the majority of the OMPs tested had no, or only very small, effects on the activity of OmpT (Figure 2c) (e.g. ∼30% increase in the initial rate of peptide cleavage in the presence of BtuB and an ∼30% decrease with OmpX, both at a 1:2.4 OmpT:OMP ratio). In marked contrast, in the presence of OmpA or tOmpA the activity of OmpT is increased by up to 2-fold (Figure 2b, d). This effect is saturated at a ∼1:1 OmpT:OmpA (or OmpT: tOmpA) ratio, suggestive of a specific 1:1 interaction between the two proteins (Figure 2d, Figure S3). Given that OmpA and tOmpA have the same effect, the enhancement of activity must be mediated via formation of a specific complex between OmpT and the β-barrel domain of OmpA. Determination of the *K*_M_ of OmpT for its peptide substrate revealed that the increased activity results from a decreased *K*_M_ in the OmpT-OmpA complex compared to OmpT alone (*K*_m_ of 179 μM ± 31 μM vs 364 μM ± 92 μM, respectively, 95% Confidence Limits), while the *V*_max_ remains unchanged (Figure 2e). To test the specificity of the observed effect, OmpA and OmpT were co-folded into DMPS, DMPC and DLPC liposomes at a 1:1 M ratio (Figure S4a, b) and OmpT activity again determined. While a ∼2.6-fold rate enhancement in OmpT activity was observed in DMPS membranes (Figure S4c), no activity was observed for OmpT in the presence or absence of OmpA in DLPC or DMPC membranes (Figure S4d), consistent with the previous reports showing that OmpT requires negatively charged lipids for activity [20,40].

The molecular origins of the observed rate enhancement were next probed by assaying OmpT activity in the presence of OmpA variants in which the extracellular loops were mutated to contain only negative residues, only positive residues or no charged residues (OmpA-Neg, OmpA-Pos and OmpA-Neut, respectively) [41] (Figure 1b) (Methods). For OmpA-Pos and OmpA-Neut the rate enhancement in OmpT activity caused by the presence of WT OmpA was no longer observed, while OmpA-Neg caused a significant, but much weaker, enhancement of OmpT activity (∼30% of that observed with WT OmpA) and which still saturates at an ∼1:1 mol/mol ratio), indicating the importance (but not sufficiency) of the presence of negatively charged residues in the loops in mediating this effect (Figure 2f). Given that OmpA-Neg is three-times less effective at enhancing OmpT activity than WT OmpA, it is possible that the precise conformation of the extracellular loops could be altered by the seven mutations in OmpA-Neg, causing this effect. To test this notion, OmpT activity was also measured in the presence of a previously-created variant of tOmpA in which short turns replace all four of its extracellular loops (OmpTran-s3) [38]. Notably, OmpTrans3 has no effect on OmpT activity, confirming the requirement for both the extracellular loops and their negative charge for the rate enhancement observed with WT OmpA (Figure 2f). Additionally, it was found that OmpA/tOmpA were unable to activate OmpT when reconstituted in separate liposomes and then mixed (Figure S5).

**Figure 3:**
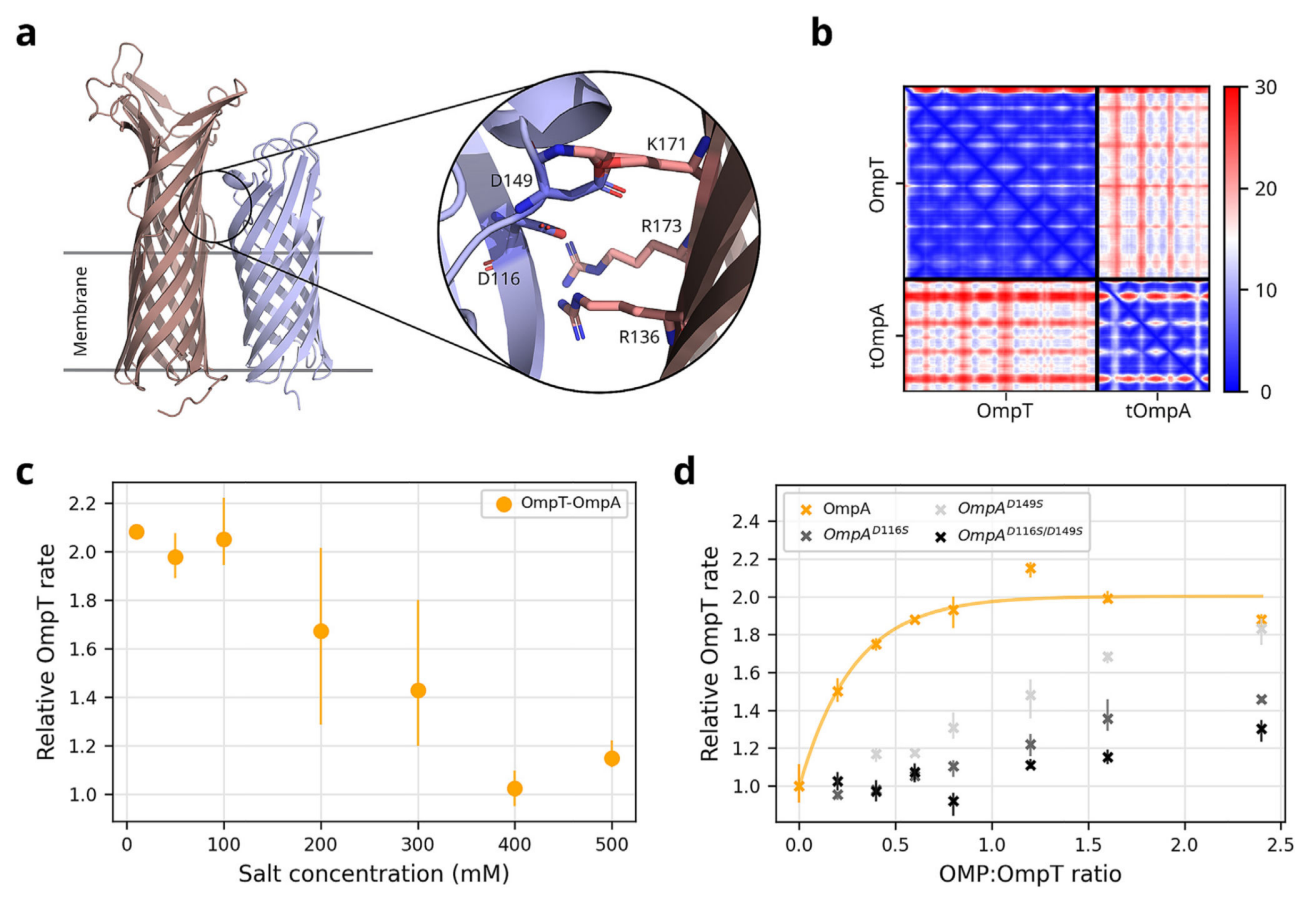
Alphafold2 prediction of OmpT–OmpA interaction. **(a)** OmpT and OmpA are predicted to interact via their β-barrel domains (note: the OmpA C-terminal domain was excluded from the prediction), with stabilising electrostatic interactions predicted to form between their extracellular loops (inset). Due to the slight tilt in the predicted OmpA position within the complex there are minimal interactions between the transmembrane domains. **(b)** PAE plot of the complex, indicating intermediate confidence predictions throughout the inter-strand region. **(c)** OmpT activity in the presence of OmpA (1:1 ratio) relative to the absence of OmpA, at increasing salt concentrations. **(d)** Initial rate of OmpT activity in the presence of increasing concentrations of OmpA^D116S^, OmpA^D149S^ and OmpA^D116S/D149S^. (**c-d** with 50 μM peptide substrate, 0.05 μM OmpT, *n* ≥ 3 in all experiments, error bars are data range).

Next, to examine the interaction surface between the β-barrels of OmpT and tOmpA, Alphafold2 was used to predict a putative dimer between the two proteins (Figure 3a, b), yielding a medium-confidence complex (inter-chain adjacent residue PAE ∼15). This complex has electrostatically-mediated extracellular loop interactions with two negatively charged residues on OmpA (D149, D116) predicted to interact with positive residues on OmpT (R136, K171, R173) (Figure 3a, inset), in agreement with the biochemical data presented above (Figure 2f). Due to the slight tilt in the predicted OmpA molecule, there are few interactions between the transmembrane domains, possibly indicating an intermediary lipid molecule. The importance of electrostatics in mediating the OmpT–OmpA interaction was experimentally validated by demonstrating that increasing amounts of NaCl could ablate OmpA’s rate enhancing effect, presumably via electrostatic shielding (Figure 3c). Finally, the single (OmpA^D116S^, OmpA^D149S^) and double (OmpA^D116S/D149S^) OmpA charge mutants suggested by the Alphafold2 prediction to mediate the OmpT–OmpA interaction were generated and their efficient *in vitro* folding confirmed (Figure S1). Assessing the rate enhancement of OmpT in the presence of these mutants demonstrated that all showed reduced ability to enhance OmpT activity (Figure 3d), with the double mutant more impaired compared to the single mutants. They also lose the ∼1:1 stoichiometry of binding observed with OmpA-WT. We conclude, therefore, that OmpA specifically enhances OmpT activity by increasing substrate affinity, via an electrostatically dominated interaction and dependent on the conformation of OmpA’s loops.

### OmpLA activity is not affected by the presence of other OMPs

OmpLA is an OMP phospholipase which strictly requires divalent cations ions for its activity [42]. While OmpLA in DMPG liposomes is stable in the absence of divalent cations for >24 h (Figure S6a), upon addition of CaCl_2_ the liposomes are rapidly degraded (as measured by dynamic light scattering (DLS) or absorbance at 510 nm, Figure S6b-d). While OmpLA activity can be measured in detergent micelles using synthetic substrates [43], its ability to degrade lipid membranes poses a challenge for measuring its activity in proteoliposomes. To address this, an assay for OmpLA activity was developed that detects the production of the free fatty acid (FFA) released upon phospholipid degradation, based on the differential fluorescence of ANS (8-anilinonaphthalene-1-sulfonic acid) which is fluorescent when bound to bovine serum albumin (BSA), but only weakly fluorescent when free in solution [44]. By exploiting the natural affinity of FFA for BSA [45,46], the relative concentration of FFA can be determined as it displaces the ANS from BSA, leading to a reduction in fluorescence. Controls in which the FFA oleic acid was added to BSA-ANS demonstrated the expected reduction in ANS fluorescence upon addition of the FFA (Figure S7a, b). Incubating BSA-ANS with DMPG-OmpLA proteoliposomes and then adding CaCl_2_ to initiate the reaction yielded reproducible activity traces revealed by the reduction in ANS fluorescence caused by the release of FFA by the enzyme (Figure S7c, d).

**Figure 4:**
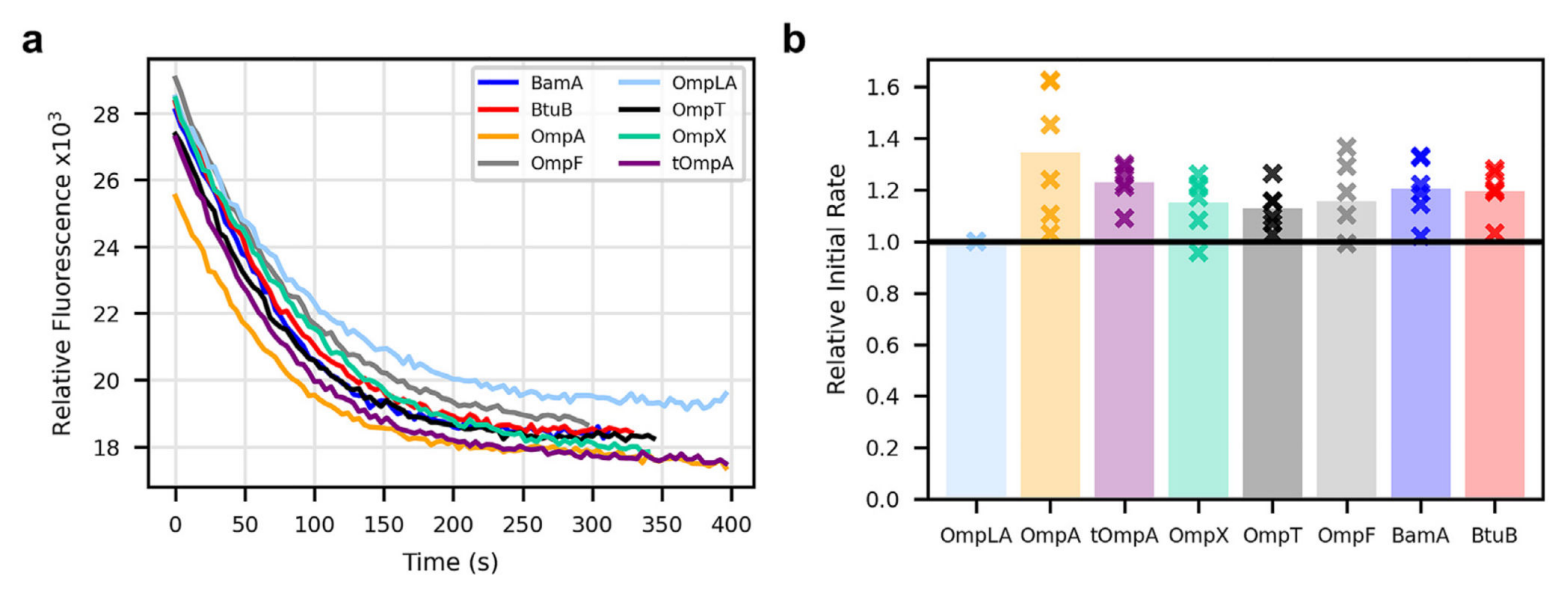
OmpLA activity is minimally affected by the presence of other OMPs. **(a)** Representative kinetic traces of OmpLA activity in the absence or presence of an equimolar concentration (0.025 μM) of other OMPs, measured by ANS fluorescence. In this assay ANS fluorescence intensity is decreased as the fatty acid products of phospholipid degradation displace ANS from BSA-ANS complexes. **(b)** Relative initial rates of OmpLA activity in the presence of other OMPs compared to OmpLA alone.

Using this ANS fluorescence assay, the phospholipase activity of OmpLA was measured in DMPG proteoliposomes in the absence or presence of a 1:1 mol/mol ratio of other OMPs (Figure 4a). Calculating the initial rates, relative to the average initial rate of OmpLA alone, show a significant, but small (∼20−30%), rate enhancement in the presence of all the OMPs (Figure 4b), with the presence of OmpA yielding the largest effect (34%). Thus, by contrast with the findings for OmpT, the activity of OmpLA is not enhanced specifically by OmpA/tOmpA. Instead, all added OMPs tested here result in a small enhancement in activity that does not saturate and hence is likely to be a non-specific effect on OmpLA enzyme activity induced by increased OMP concentrations in the liposomes.

### OmpA specifically reduces PagP activity

PagP, an OMP acyltransferase, was the only tested OMP that failed to fold into DMPG membranes under the conditions employed here for the set of OMPs, consistent with previous reports that PagP folds into bilayers with poor efficiency in low concentrations of urea (0.5 M urea was used in the assays herein), presumably because the protein aggregates before it can successfully fold and insert into the membrane [39]. To circumvent this, and again following literature precedents [39], PagP was found to fold efficiently into 100 nm DMPG liposomes in 6 M urea, confirmed by a shift in the intrinsic fluorescence peak maximum and far UV CD spectra which showed β-sheet formation indicating successful folding under these conditions (Figure S8a, b). Once folded into the liposomes, PagP remains stably folded when the concentration of urea is decreased to 2 M, conditions which also enable successful folding of tOmpA, OmpA and OmpX (Figure S9). Hence, by folding PagP into DMPG liposomes in 6 M urea, followed by dilution into 2 M urea, proteoliposomes containing folded PagP co-assembled with tOmpA, OmpA or OmpX could be prepared.

**Figure 5:**
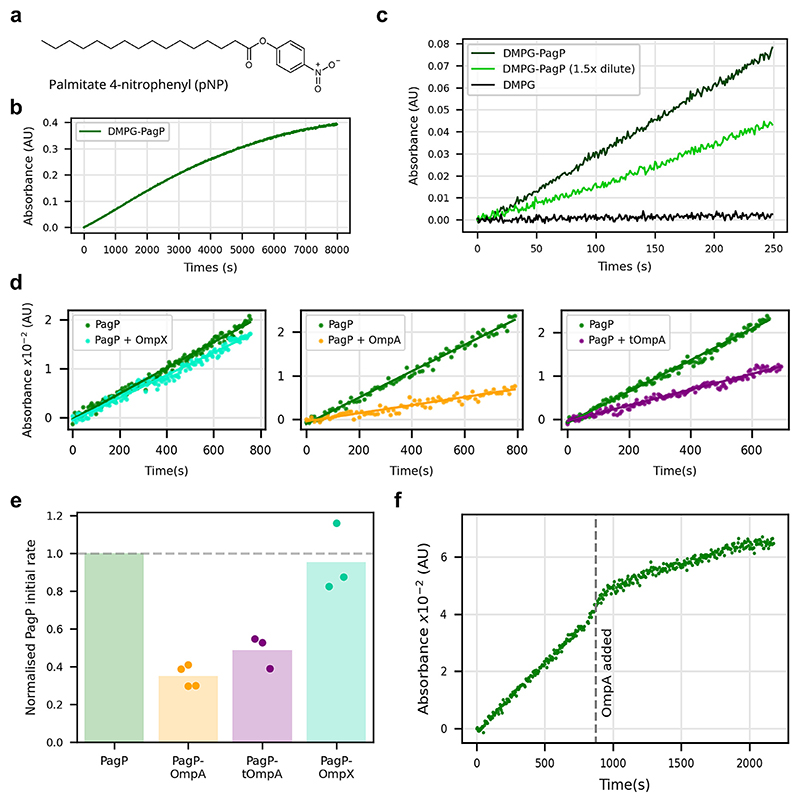
OmpA specifically decreases PagP activity. **(a)** Structure of palmitate 4-nitrophenyl (pNP), a PagP substrate. **(b)** Activity of PagP in DMPG proteoliposomes to which pNP has been delivered using MβCD. **(c)** PagP activity at different enzyme concentrations (different proteoliposome LPR, PagP at 1 μM and 0.66 μM) and in empty DMPG liposomes. **(d)** Activity of PagP in the presence of 1:1 M ratio of OmpX, OmpA or tOmpA and **(e)** the initial rates normalised to that of PagP alone (0.25 μM PagP). **(f)** The activity of PagP is reduced when equimolar OmpA is folded into the proteoliposomes (dashed line indicates time of addition of OmpA, 2% total volume change upon addition).

A colorimetric activity assay for PagP activity in detergent micelles has previously been reported [39], in which the cleavage of the palmitate group in the synthetic substrate palmitate-4-nitrophenol (pNP) releases free, yellow coloured, nitrophenol (Figure 5a). To develop this assay for use with PagP-containing proteoliposomes, the pNP substrate must first be delivered to the membranes to begin the reaction. Cyclodextrins have been used previously to deliver hydrophobic lipid-like molecules to bilayers [47]. Accordingly, the ability of methyl-β-cyclodextrin (MβCD) to rapidly deliver pNP to liposomes as a substrate for PagP activity was tested. The results revealed the successful and rapid (within 5 sec) delivery of pNP into DMPG liposomes (Figure 5a-c), providing a method to assay PagP activity in proteoliposomes. Controls showed that the liposomes are unaffected by MβCD at the concentration used (2-fold molar excess over the lipid concentration) (measured using DLS) (Figure S10a; and that the phase transition temperature of DMPG membranes incubated with MβCD-pNP is increased in the presence of pNP, but not when incubated with MβCD alone (using the fluorescent reporter laurdan) (Methods), indicating successful and rapid substrate delivery (Figure S10b-d). Thus, MβCD facilitates the rapid delivery of the pNP PagP substrate into liposome bilayers, enabling enzyme kinetics to be measured (Figure S10d, e).

PagP activity in DMPG proteoliposomes with or without an ∼1:1 ratio of OmpX, OmpA or tOmpA is shown in Figure 5d. While no difference in initial rate is observed in the presence of OmpX, in the presence of OmpA and tOmpA a clear decrease PagP activity is observed (to ∼35% and ∼45%, respectively, Figure 5e). The effect of OmpA on PagP activity was demonstrated by folding OmpA into the liposomes during measurement of pNP hydrolysis (folding time ∼60 s (Figure S1a)) and observing the concomitant decrease in PagP enzyme activity (Figure 5f). Despite the effect of (t)OmpA on PagP activity, Alphafold2 failed to predict a confident interaction between the proteins (Figure S11). Nonetheless, by these activity assays it is clear that OmpA can regulate the activity of different OMP enzymes, increasing the activity of OmpT, decreasing that of PagP, and having little effect on OmpLA.

### Prediction of OMP–OMP interactions across the entire E. coli OMPome

To search systematically for other potential OMP–OMP interactions in the OM, Alphafold2 predictions were made of potential pairwise interactions of the seven most abundant OMPs (estimated > 2% count of the OM protein content; OmpA, OmpC, OmpF, OmpX, OmpT, MipA, Tsx) with the 59 confirmed *E. coli* OMPs. To ensure that interactions are not solely via the soluble, C-terminal domain of OmpA, interactions with tOmpA were also considered.

**Figure 6:**
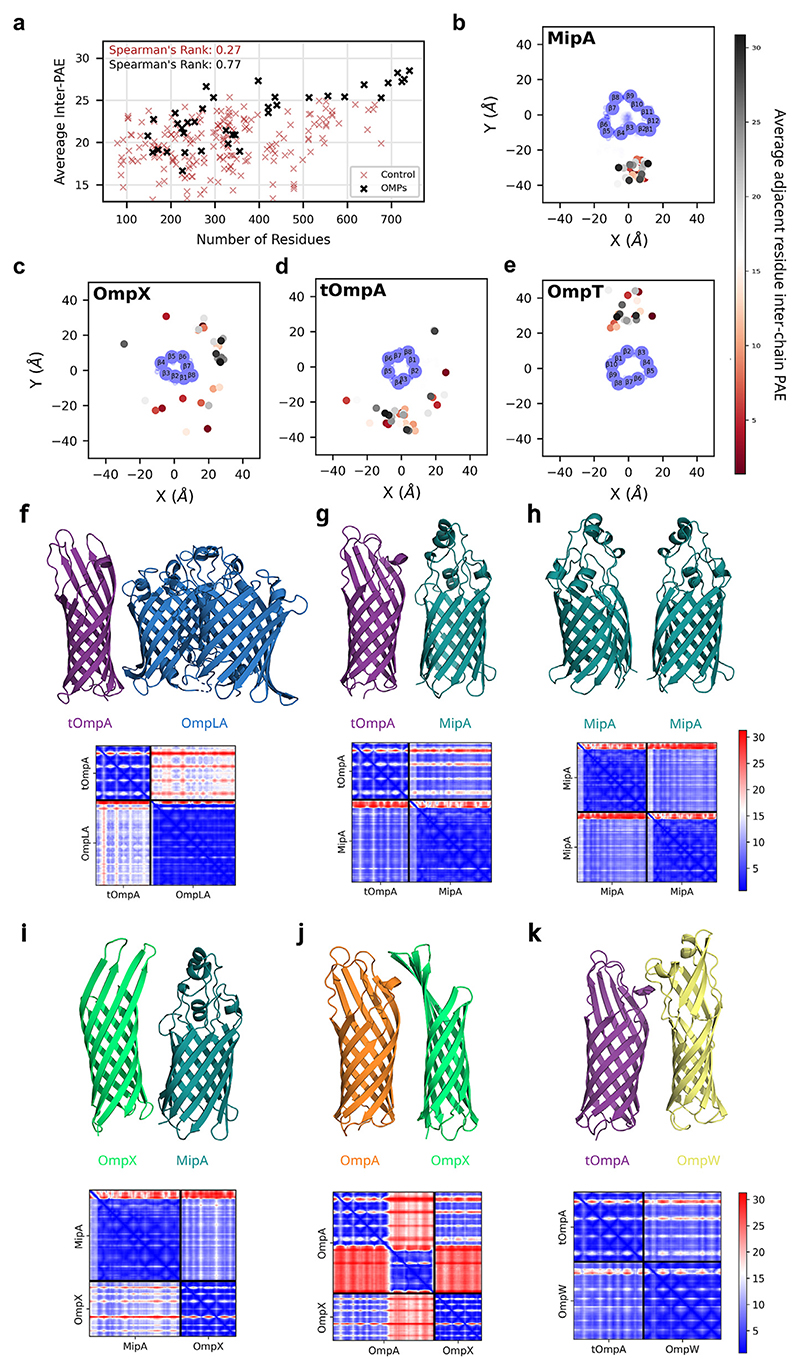
AlphaFold2 predictions of OMP–OMP interactions. **(a)** Considering the average residue-adjacent inter-chain PAE from all OMPs to the most abundant OMPs reveals that smaller OMPs are consistently predicted to be more promiscuous interactors (black), in contrast to a control dataset of protein pair predictions that shows a minimal correlation between protein size and PAE (pink). **(b-e**) Angular distributions of the centre of mass of the transmembrane domains of predictions around the abundant OMPs, coloured by inter-chain PAE (aligned abundant OMP in blue with labelled β-strands). **(f-k)** A subset of the predicted high confidence interactors (tOmpA-OmpLA, tOmpA-MipA, MipA-MipA, MipA-OmpX, OmpA-OmpX, tOmpA-OmpW, respectively) with PAE plots (blue indicates high confidence and red low confidence). In **(f)** tOmpA-OmpLA was predicted with a single copy of OmpLA, a second copy was then added for display to show the dimer interface. In **(j)** the C-terminal domain of OmpA is not shown for clarity (both domains are in the PAE plot).

Following quality filtering (pLLDT > 80), the average adjacent-residue (Cα-Cα cutoff of 1.2 nm) inter-chain PAE was calculated for each prediction (Figure S11). As well as identifying known interactions, such as those in trimeric porins, this analysis highlighted multiple unexpected, medium−high probability interactions (PAE < 15), mostly involving the smaller OMPs, tOmpA, OmpX and MipA. Indeed, comparing the distribution of PAEs over all predicted complexes indicates that smaller OMPs are more likely to be predicted as promiscuous interactors, (Figure 6a), while a non-OMP control dataset indicates prediction size generally minimally correlates with PAE. The binding surfaces of each of the abundant OMPs was then assessed by identifying the distribution of OMPs around each protein (Figure 6b–e, Figure S12). The results yielded a range of binding patterns with MipA and OmpT predicted to have a preferred, single binding site, while the other OMPs, including tOmpA and OmpX, are predicted to interact at a more diverse range of sites.

Some of the higher confidence Alphafold2 predictions are shown with their PAE plots in Figure 6f–k (see also Figure S13). As noted above, they are enriched in (t)OmpA, MipA and OmpX. Interestingly, a high confidence interaction is found for tOmpA-OmpLA_dimer_ (away from the OmpLA dimeriation interface, Figure 6f) indicating that, despite lacking a significant or specific activity change, the transmembrane domain of OmpA likely does interact with OmpLA. Possible utility of other complexes can be speculated based on functional correlations. For example both OmpA and MipA interact with peptidoglycan (tOmpA-MipA, Figure 6g) while a MipA dimer may more effectively support assembly of the hypothesised peptidoglycan hydrolase complex [48] (MipA-MipA, Figure 6h). However, some complexes (for example, tOmpA-OmpW, OmpX-MipA, OmpA-OmpX, Figure 6i-k) have no clear functional implications based on known protein activities, possibly reflecting a role for the promiscuous binding of these proteins to form and stabilise OMP islands, with both the transmembrane and extracellular regions forming major interaction sites (Figure S13). Together, this analysis suggests that specific OMPs, generally smaller ones, are important for mediating OMP–OMP interactions in the OM, and coupled with the above biochemical data, highlights the promiscuity of the β-barrel of OmpA, which forms many different, yet precise, interaction partners which can specifically modulate function.

### LPS and OmpA cooperatively activate OmpT

**Figure 7:**
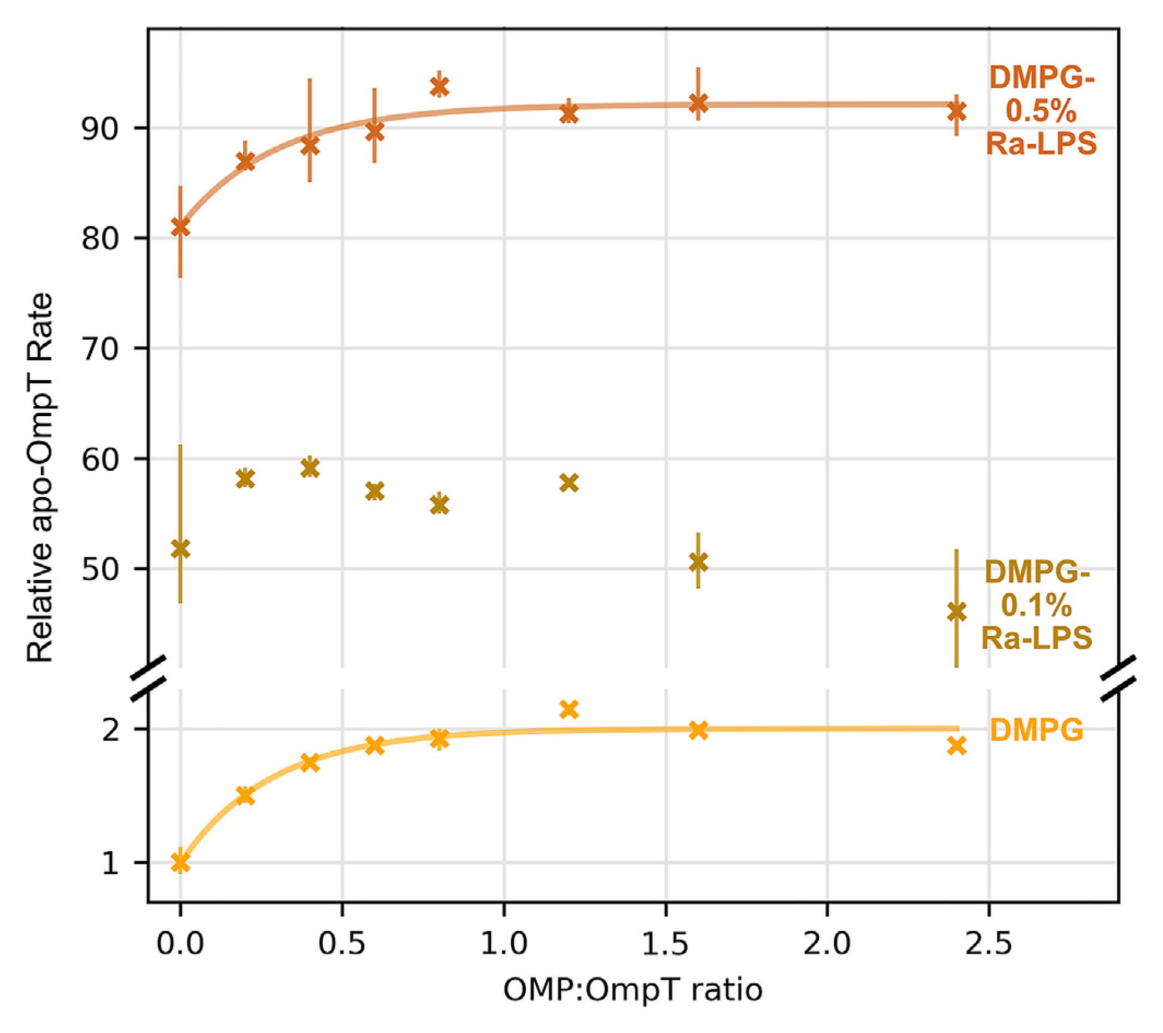
LPS and OmpA activate OmpT. Initial rate of OmpT activity in DMPG liposomes supplemented with none, 0.1% or 0.5% Ra-LPS and in the presence of increasing concentrations of OmpA. The data for DMPG liposomes and DMPG-0.5% Ra-LPS are fitted to a single-phase exponential. (50 μM peptide substrate, 0.05 μM OmpT, *n* ≥ 3 in all experiments, error bars are data range).

OmpT has an established interaction with OM outer leaflet lipid LPS that is known to dramatically increase its activity [8,9]. Given this, we assayed the activity of OmpT in the presence of both OmpA and LPS by generating proteoliposomes including 0.1% (mol/mol) or 0.5% (mol/mol) Ra-LPS in the DMPG liposome background. In the presence of LPS alone (0.5% mol/mol Ra-LPS) OmpT is activated by ∼80-fold, and with an excess of OmpA ∼90-fold, relative to apo-OmpT activity (Figure 7). Similar to the results obtained in the absence of LPS, the rate enhancing effects of OmpA in the 0.5% Ra-LPA-containing proteoliposomes reaches a plateau at ∼1:1 OmpT:OmpA, indicative of a specific 1:1 interaction between the two proteins. It is intriguing that in absolute terms, the magnitude of the rate enhancement by OmpA increases ∼5-times in the presence of LPS, suggesting that LPS and OmpA are able to cooperatively activate OmpT. In contrast, the 0.1% LPS-containing proteolipo-somes support only ∼50-fold activity enhancement which diminishes at higher OmpA ratios (Figure 7), likely due to OmpA’s known ability also to bind LPS [10,49], which could compete with OmpT and thus prevent a productive OmpT-LPS interaction. Indeed, LPS is limited in the 0.1% proteoliposomes, with an approximate OmpT:LPS ratio of only 1:3. Combined, these results indicate that while both OmpA and LPS are sufficient to activate OmpT alone, maximal activity is observed in the presence of both interactions.

## Discussion

The native OM is crowded with OMPs in a sparse lipid context, with many OMPs known to cluster, even in lipid-rich *in vitro* contexts [3,4,10,11]. However, while these features mean that modulation of OMP activity via specific OMP–OMP interactions is biochemically plausible and evolutionarily expected [50,51], it has not previously been demonstrated. Here we find that interactions with OmpA can modulate enzyme activity of different OMPs in a manner that appears specific (the effect is not observed upon the addition of other common OMPs). For OmpT, the presence of OmpA specifically increases enzyme activity, with the effect saturating at a 1:1 OmpA;OmpT ratio. By contrast, OmpA binding causes a decrease in the enzyme activity of PagP, while for OmpLA there is little effect on its activity. The observed rate modulations are specific to OmpA, and are not seen upon the addition of other common OMPs. While OmpA and the trimeric porins OmpF/C together make up about two thirds of the OM’s protein content in *E. coli* [18], unlike the trimeric porins whose proportions can change depending on environmental conditions [52], OmpA is consistently highly expressed allowing it to constitutively interact with other OMPs [53]. Furthermore, the porins partially segregate into arrays in the membrane [4], making them less available to interact with other OMPs, and their loops are partly sequestered in order to maintain their trimeric state and pore structure [54], leading to additional selective pressures which limit options for evolving other protein-protein interactions. In contrast, OmpA is evenly dispersed throughout the OM [55] and amongst other OMPs [56] (although possibly as a dimer [57]) and functions attributed to its extracellular loops would not preclude them from forming OMP–OMP interactions [58]. It is interesting that the rate enhancement of OmpT caused by the presence of OmpA is explicitly mediated by the charge in the OmpA loops and not its barrel, suggesting additional evolutionary constraints on the barrel sequences [59,60], which lim its their malleability to develop new interactions. Recent studies have shown that the interaction of the OmpA C-terminal domain with peptidoglycan is functionally decoupled from its β-barrel domain, with the transmembrane domain providing only an anchor, suggesting it could evolve distinct roles within the OM [32]. Together, these considerations suggest that OmpA is the ideal protein to have evolved the additional functionality of mediating specific OMP–OMP interactions that can modulate function, as identified here.

OmpT is a constitutively active protease with a strong preference for positively charged substrates [61], and its role is to protects cells from peptide threats, typically cationic host antimicrobial peptides. Enhancing the activity of OmpT by its interaction with OmpA in the OM would thus offer a greater protection to the cell against these threats. Specific OmpA binding by OmpT would also support OmpT function by potential utilisation of the negative charges in OmpA’s loops to enhance binding of its positively-charged antimicrobial targets to the enzyme active site, in competition with the strong negative charge of the LPS layer itself. Notably, the OmpT activation by LPS and OmpA are additive, hinting at how multiple elements of the complex environment of the OM may come together to modulate OMP function.

In contrast to its effect on OmpT activity, PagP activity is decreased by OmpA, but the reasons for this remain obscure. While phospholipid substrate accessibility is a known regulator of PagP activity [62] (and OmpA occlusion of this site could explain the reduction in activity), given the low diffusivity in the OM, it is unclear how PagP can access sufficient LPS to alter global membrane properties, especially given the precise substrate approach needed for its activity [63]. However, given its LPS substrate requirement, it is likely that PagP could localise to LPS-rich regions of the membrane. Any PagP molecules that might become mislocalised to OMP islands would thus be catalytically slowed by interacting with OmpA, possibly helping to prevent disruption of the tightly packed LPS-OMP networks. Alternatively, OmpA and PagP have both been implicated in OM vesicle (OMV) formation via control of the OM-peptidoglycan linkage [64] and modulating hepta-acylation of LPS, respectively, with increased acylation enhancing OMV formation [65]. This suggests that a PagP and OmpA interaction could be part of a regulatory mechanism governing OMV formation, although further work is needed to understand the physiological role of such an interaction.

While we show here that OmpA modulates the enzymatic activities of OmpT and PagP, the activity of OmpLA was not specifically modulated by any of the OMPs tested under the conditions employed. Thus, the effects of OmpA binding on OmpT and PagP activity are not a generically evolved feature of OMPs but rather appear to be specific for their functional niche. While retaining a β-barrel core, OmpT, PagP and OmpLA are structurally and dynamically distinct with highly diverse loopsand structural ensembles [66,67] (Figure S14), making it challenging to interpret exactly which features of the different proteins mediates the modulatory effects. While OMP−OMP interaction do not always induce functional modulation, the Alphafold modelling presented here indicates that there is a higher level of co-evolution between the smaller OMPs and the general OMPome than for larger OMPs, and a tendency to have interactions predicted over more of their surface area, suggesting a role for the smaller OMPs, in general, in mediating OMP clustering. The most abundant OMPs, which will have the strongest evolutionary pressure to be general interactors, are typically small (two 8-stranded, two 10-stranded and two 16-stranded) allowing them to better pack inside OMP islands and minimise possible membrane defects, especially under lipid-limiting conditions. Indeed, the ability to interact with multiple partners and hence form part of the glue holding OMP islands together is likely to be a specifically evolved function of these OMPs, and agrees with previous simulation data [3,10]. Indeed, this function has recently been shown for OmpA, which is required for the formation of OMP-lattices in the OM [32].

In summary, the results presented here provide new insights into OM organisation and the role of OmpA specifically in modulating the enzyme function of OMPs in the OM. We also exemplify DMPG as a useful synthetic membrane competent to mediate efficient and controllable folding of OMPs and OMP variants, critical for their *in vitro* characterisation and for biotechnology where many functionally designed proteins are based on transmembrane β-barrels [38,68]. Specific modulatory OMP–OMP interactions are identified between OmpA and three different enzymes of the OM, while Alphafold modelling suggests that the small abundant OMPs are the most promiscuous OMP binders, potentially facilitating OMP cluster formation in the OM - together suggesting interactions to further explore *in vivo*. Although many mechanistic details remain to be revealed, the results presented provide a glimpse of the complex functional interaction networks that could occur in the protein-crowded OM and indicate the evolutionary exploitation of common interaction interfaces to ensure optimal cell fitness.

## Methods

### Molecular biology and plasmids

All OMPs and OMP variants were expressed as the untagged, mature sequences as cytoplasmic inclusion bodies. Plasmids for OmpA, tOmpA (OmpA_1_-_171_), PagP, BamA and OmpLA were obtained from Karen Fleming, John Hopkins University. The OmpTrans3 plasmid was obtained from David Baker, University of Washington. OmpF, OmpT, BtuB and MipA were amplified by colony PCR from K12 *E. coli* and restriction ligated into a pET11a backbone (pBAD for BtuB). OmpX and OmpA-Pos (D41S, E53N, E89V, D126S, D137S, D180S and D189S), OmpA-Neg (R81S, K85T, K94S, R124S, K128G, K134S and R177S) and OmpA-Neut (combined mutations of OpA-Neg and OmpA-Pos) genes were ordered (OmpX: Twist Biosciences, OmpA variants: GeneWhizz) and restriction ligated into a pET11a backbone. Plasmids for OmpA^D116S^, OmpA^D149S^ and OmpA^D116S/D149S^ were generated by Q5 site directed mutagenesis and confirmed with sequencing.

### Protein purification

Competent BL21(DE3) *E. coli* cells were transformed with the relevant plasmid, grown overnight at 37 °C on agar plates, and a single colony used to inoculate an overnight starter culture (37 °C, 200 r.p.m.). Then, 5 ml culture was added to 500 ml LB, grown to an *A*_600_ of ∼0.6 and protein expression induced overnight with 1 mM IPTG (isopropylthiogalactoside). The cells were harvested (5,000 g, 15 min, 4 °C) and then resuspended in 20 ml buffer (50 mM Tris−HCl, pH8.0, 5 mM EDTA, 1 mM phenylmethylsulfonyl fluoride, 2 mM benzamide) and lysed via sonication. Following centrifugation (25,000*g*, 30 min, 4 °C), the pellet was resuspended in 20 ml buffer (50 mM Tris−HCl, pH8.0, 2% (v/v) Triton-X-100) and incubated for 1 h (room temperature, 50 r.p.m.). Following centrifugation (25,000*g*, 30 min, 4 °C), the supernatant and cell debris were removed from the pelleted inclusion body. The inclusion bodies were washed twice by resuspending in 50 mM Tris−HCl (pH8.0) and incubating for 1 h (room temperature, 50 r.p.m.) before pelleting by centrifugation (25,000*g*, 30 min, 4 °C). The inclusion body pellet was then solubilised in 25 mM Tris−HCl and 6 M Gdn-HCl (pH 8.0) for 1 h (50 r.p.m. stirring), and following a final centrifugation (25,000*g*, 30 min, 4 °C), the supernatant was loaded onto a Superdex 75 HiLoad 26/60 size-exclusion chromatography column (GE Healthcare), equilibrated in 25 mM Tris−HCl (pH 8.0) and 6 M guanidinium-HCl. Protein fractions were collected and concentrated to ∼100 μM (Vivaspin concentrators) and flash-frozen for storage at −80 °C. Before folding, proteins were buffer-exchanged into Tris-buffered saline (20 mM Tris−HCl, 100 mM NaCl, pH8.0) and 8 M urea using 0.5 ml Zeba spin desalting columns with a molecular weight cut-off of 7 kDa (Thermo Fisher Scientific).

### Liposome preparation

The required amount of resuspended DMPG (dimyristoylphosphatidylglycerol, Avanti polar lipids, in 1:4 MeOH:chloroform), supplemented with Ra-LPS (Sigma L9641) as required, was dried to a thin film in a glass vial and desiccated overnight. Following resuspension to a stock concentration of 40 mM in buffer (20 mM Tris−HCl (pH 8.5), 50 mM NaCl), the lipids were freeze−thaw cycled using liquid N_2_ and an ∼50 °C water bath and then extruded through 100 nm nucleopore polycarbonate track-etched membranes (Whatman, Avanti extruder) at 35−40 °C (>10 °C higher than the lipid *T*_m_). For salt titration experiments the proteoliposome buffer NaCl concentration was adjusted as necessary.

### OMP folding

Folding kinetics of OMPs were measured using a QuantaMaster fluorimeter (Photon Technology International (PTI)), including a peltier-controlled temperature unit, controlled by FelixGX software (v4.3). Excitation/emission wavelengths of 280/335 nm were used. Unfolded OMPs (8 M urea, 25 mM Tris−HCl, pH 8.5) were rapidly diluted to a final concentration of 0.05-0.2 μM OMP (depending on number of Trp residues) and 0.5 M urea in the presence of DMPG liposomes (final lipid/protein ratio of 800:1 (mol/mol)) in 20 mM Tris−HCl (pH 8.5) and 100 mM NaCl at 24 °C. Emission spectra of the unfolded protein (in 8 M urea) and the post-folding reaction were also collected (excitation 280 nm, emission 300−400 nm) at 24 °C. Experiments in 2 M urea were conducted identically, but with a final urea concentration of 2 M.

### SDS-PAGE gels

Samples were mixed in a ratio of 1:3 with loading dye (50 mM Tris−HCl, pH6.8, 6% (w/v) SDS, 0.3% (w/v) bromophenol blue, 40% (v/v) glycerol), boiled if required (>10 min, 100 °C) and ∼14 μl sample loaded onto the gel. Precision Plus Protein Dual Xtra Standards (BioRad) were used as molecular weight markers. Gels were 15% Tris-tricine containing 0.1% (w/v) SDS and 1 M Tris−HCl at pH 8.45 with 13.3% (v/v) glycerol included in the resolving layer. The cathode buffer consisted of 100 mM Tris−HCl, 100 mM tricine and 0.1% (w/v) SDS (pH8.25) and the anode buffer comprised 200 mM Tris−HCl (pH8.9). Electrophoresis was conducted with constant currents of 30 mA (stacking) and 60 mA (resolving). Following staining (InstantBlue Coomassie, Abcam), the gels were imaged using a Q9 alliance imaging system (Uvitec) and densitometric analysis was performed using ImageJ. For sensitive OMPs (OmpLA, BamA, OmpT), gels were made without SDS, 4× loading dye with 1% SDS, and run overnight at 4 °C (10 mA).

### OmpT:OMP proteoliposome preparation and activity assays

OmpT proteoliposomes were generated by folding of the required OMPs into DMPG liposomes, prepared in a 20 mM Tris−HCl pH 8.5, 50 mM NaCl buffer. DMPG OmpT proteoliposomes were prepared by adding 1 μM OmpT (unfolded in 8 M urea, final concentration of urea for folding 0.5 M) to 6 mM DMPG at 24 °C mfor ∼30 min (i.e. 1:6000 mol/mol LPR). The required molar ratio of each folded OMP (typically 0, 0.2, 0.4, 0.6, 0.8, 1.2, 1.6, 2.4 times OmpT) were added to the OmpT-DMPG proteoliposomes (or, for *in trans* mixing experiment, to empty DMPG liposomes) with a 2-fold dilution of OmpT-DMPG, maintaining a final urea concentration at 0.5 M, and incubated at 24 °C for ∼30 min. For activity assays, the refolded OmpT-OMP mixture was diluted 10-times (final OmpT concentration of 0.05 μM) with the synthetic substrate ARRAY (Abz-Ala-Arg-Arg-Ala-Tyr(NO_2_)-NH_2_, Protein Synthetics) (final ARRAY concentration of 50 μM). Samples were measured on a platereader (BMG Clariostar, 325/430 nm excitation/emission) using 96-well plates (Corning Costar, black plates with transparent bottoms) and 100 μl volume, with fluorescent readings every 90 s and 10 s of gentle shaking (100 rpm) prior to each reading. Initial rates were extracted by fitting to the linear region of the activity trace. For determining *K*_M_ and *V*_max_: 5 μM, 25 μM, 50 μM, 100 μM, 150 μM, 250 μM, 500 μM or 1000 μM ARRAY was added to preformed DMPG proteoliposomes containing equimolar OmpT and OmpA and OmpT activity was measured. The initial rate was determined from the linear region of the activity trace and fitted to the Michaelis-Menten equation. Errors were estimated by pooling all data and determining the 95% confidence limits by bootstrapping. For activity measurements in different lipids: OmpT or 1:1 OmpT–OmpA were first refolded into DMPC or DLPC liposomes (6000:1 LPR) by incubation overnight at 24 °C, or for DMPS liposomes by incubation at 35 °C for 30 min, and then OmpT activity was measured as described above. For salt titration experiments the proteoliposome reaction buffer NaCl concentration was adjusted as necessary.

### Dynamic light scattering (DLS)

DLS was performed on a Wyatt miniDawnTreos® system (equipped with an additional DLS detector). Filtered (0.22 μm) buffer was used to obtain ∼5 min baselines before and after sample injection and system was washed with 1 M nitric acid and 18X-H_2_O after each run, followed by 1 mL of buffer. Proteoliposomes were diluted to ∼5–10 μg/ml lipid and 300 μl injected. Correlation curves were analysed using the Astra 6.0.3® software, by regularisation.

### OmpLA activity assays

OmpLA and OmpLA + OMP (always 1:1 ratio) DMPG proteoliposomes were prepared identically to OmpT and OmpT + OMP proteoliposomes. For DLS time courses, the reaction was initiated by addition of a 10-fold molar excess of CaCl_2_ (compared to OmpLA), and the reaction was quenched at each time point with a 10-times molar excess (compared to CaCl_2_) of EDTA. Light scattering of OmpLA proteoliposomes was performed with a final liposome concentration of 1 mM (OmpLA LPR of 6000:1). The reaction was initialised by addition of 10-times molar excess of CaCl_2_. Reactions were measured in paired (i.e. baseline and reaction) QS quartz cuvettes on a room temperature spectrophotometer monitoring the *A*_560_ every 1 s.

The fluorescence of ANS (200 μM), ANS + BSA (20 μM, 1 μM) and ANS + BSA + oleic acid (20 μM, 1 μM and 0.1% (v/v), respectively) was measured on a Horiba PTI with 377/470 nm excitation/emission wavelengths at 30 °C. Relative concentrations of BSA and ANS to generate a maximal response upon the addition of oleic acid were optimised. OmpLA activity was measured with 9 μM ANS, 0.5 μM BSA, 0.02 μM OmpLA in DMPG liposomes at a 6000:1 LPR. The reaction was initialised by the addition of 1 mM CaCl_2_. Platereader assays were performed on a Fluostar Omega (BMG Labtech, 430 nm filter) in 96 well plate (Corning Costar, black plates with transparent bottoms) with final concentrations of 9 μM ANS, 0.5 μM BSA, 150 μM DMPG, 0.025 μM OmpLA (LPR 12000:1); 1 μM CaC* was added to start the reaction; ∼10 s reaction deadtime. Samples were measured at 30 °C, with a 1:1 ratio of OmpLA:OMP, with measurements every 4 s. Following measurements, the initial activity was determined from the linear region of the activity trace and the *T*_50_ as the half-maximum intensity drop.

### PagP refolding

PagP refolding into DMPG (6000:1 LPR) was performed overnight in 6 M urea at 24 °C in 20 mM Tris−HCl pH 8.5, 50 mM NaCl buffer. CD spectra were measured in 1 mM QS quartz cuvettes using a Chirascan plus CD spectrometer (Applied Photophysics) with a bandwidth of 2.5 nm and adaptive sampling. Three scans were averaged between 260 nm and the lowest usable wavelength (dependent on urea concentration). PagP conformation was measured by far UV CD using 6 μM PagP in DMPG liposomes at 400:1 LPR, or unfolded in 8 M urea. PagP refolding into DDM was performed similarly, but in the presence of 2 M urea.

### PagP activity assay

#### Assay validation

MβCD-pNP (methyl-β-cyclodextrin, palmitate 4-nitrophenyl) complexes were generated by solubilising pNP in water at 70 °C, and then adding MβCD (200 mM stock) and rapidly mixing the sample at room temperature in a 2:1 M ratio of MβCD:pNP. Lipid transition temperatures were measured by laurdan fluorescence using a method adapted from [69]. Briefly, DMSO dissolved laurdan, was added to liposome samples at 3200:1 mol/mol (lipid:laurdan) and a final DMSO concentration of 0.1% (v/v). Liposomes were incubated near their transition temperature overnight. Laurdan fluorescence was detected using a PTI fluorimeter (excitation/emission 340 nm and 440/490 nm, respectively), and fluorescence measured at 0.5 °C intervals from 18 °C to 29 °C. General Polarisation (GP) was determined from the emission intensity (I) using the equation: GP = (*I*_440_ − *I*_490_)/(*I*_440_ + *I*_490_). Midpoints were determined by numerical differentiation. Time resolved phase transition changes were measured on a PTI fluorimeter twice, either recording at 440 nm or 490 nm, on different samples. A 30 s baseline was measured and then, following addition and mixing of MβCD or MβCD-pNP (5 s dead time), an additional 30 s or 60 s was recorded.

#### For PagP activity

A stock of 1 μM pNP in a 1:2 M ratio with MβCD was prepared. PagP was refolded into DMPG liposomes to a final concentration of 1 μM (6000:1 LPR) overnight as described above, then diluted three times, incubated at room temperature for 10 min and centrifuged at 5000g for 5 min. Reactions were measured in paired (i.e. baseline and reaction) QS quartz cuvettes on a spectrophotometer at room temperature. The reaction was initialised by adding pNP to a final concentration of 100 μM and rapidly mixing, and recording the *A*_410_ every 1 s for ∼1000 s. For DMPG-PagP activity measurements, PagP was refolded to a final concentration of 0.66 μM. For DDM-PagP reactions all concentrations were 10-times less (i.e. final PagP concentration of 0.033 μM, initial pNP concentration of 10 μM) and conducted in 20 μM Tris−HCl pH 8.5, 50 mM NaCl, 0.05% (v/v) DDM. Following measurements, the initial activity was determined from the linear region of the activity trace. Unfolded OmpA, OmpX and tOmpA were diluted to a final concentration of 0.5 μM in 2 M urea and immediately added to DMPG-PagP proteoliposomes at a 1:1 M ratio with PagP by incubating at 24 °C for 30 min (i.e. final urea concentration of 2 M). Mid-reaction addition of OmpA was performed at ∼22 °C in the spectrophotometer, and the sample rapidly mixed immediately after OmpA addition.

### MipA characterisation

MipA bandshift on cold SDS PAGE was tested by analysing 1.5 μM DMPG-refolded MipA (with or without boiling) by SDS-PAGE at room temperature. Far UV CD spectra were acquired in 1 μM QS quartz cuvettes using a Chirascan plus CD spectrometer (Applied Photophysics) with bandwidth of 2.5 nm and adaptive sampling. Three scans were averaged between 260 nm and the lowest usable wavelength (dependent on urea concentration). MipA was analysed at a final concentration of 6 μM in 0.05% (v/v) DDM, DMPG liposomes at 400:1 LPR, or unfolded in 8 M urea.

### Bioinformatics and Alphafold

All Alphafold predictions were performed using a local installation of Alphafold2-multimer (v3) using the reduced sequence databases, without final energy minimisation and generating five predictions. Predictions were processed via filtering on pLDDT (>80). PAE of interacting residues was defined as average adjacent-residue (Cα−Cα cutoff of 1.2 nm) between residues of different chains. Angular distributions were determined by aligning predictions against a reference of the primary OMP (which was itself aligned to have the *z*-axis down its barrel centre) and then determining the centre of mass of the transmembrane regions of each secondary OMP. For interaction location analysis: interacting residues on different chains were identified using their Caαs with a threshold of 8 A, and classified as transmembrane or extracellular using the membrane region estimated by the program immers [70] for the primary monomer. For the strand vs residue adjacent inter-chain PAE analysis: the number of strands per OMP is known in each case. The control dataset was pair complex predictions of BamA’s POTRA1 with all periplasmic proteins.

## Supplementary Material

supporting data, tables and figures

Appendix

## Data Availability

Source data files containing fluorescence folding traces, raw activity data for all assays, gel images, DLS and Alphafold prediction files are freely available at the University of Leeds Data Repository (https://doi.org/10.5518/1667).
