# Supplementary material for: OmpA Specifically Modulates the Activity of Enzymes that Reside in the Crowded Bacterial Outer Membrane
Source: J Mol Biol. Author manuscript; Available in PMC 2025 Jul 30. (PMC7617971; doi:10.1016/j.jmb.2025.169346)
Supplement: supporting data, tables and figures [file EMS207352-supplement-supporting_data__tables_and_figures.pdf]

Supplementary Figures

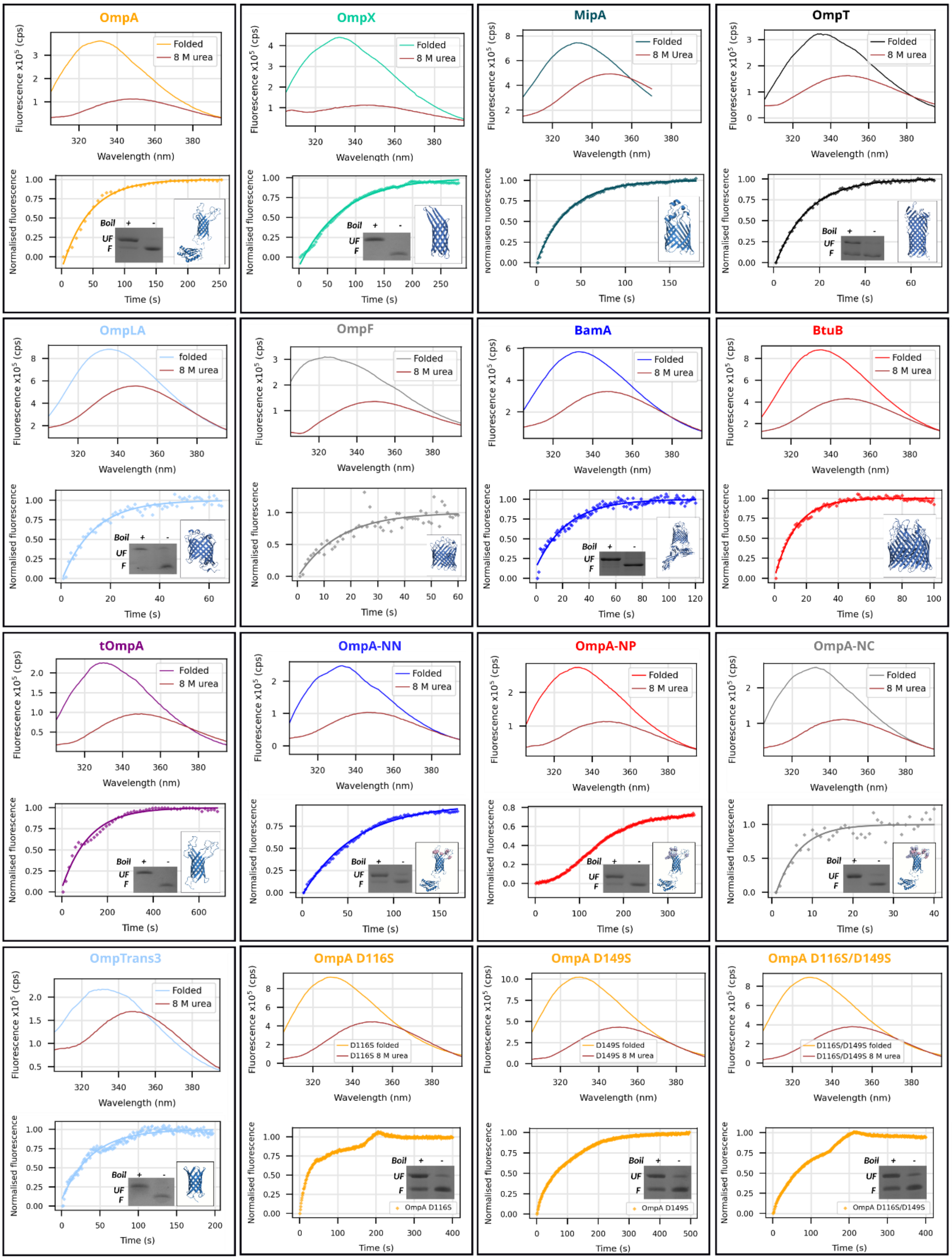

Supplementary Figure 1: OMPs rapidly and efficiently fold into DMPG. End-point fluorescence emission spectra (top) and kinetics of folding measured using intrinsic fluorescence (lower) for all natural OMPs and OmpA variants used in this study (excluding PagP, see **Supplementary Figure 7**). Where possible kinetic data are fitted to a single exponential (solid line). Folding was conducted at an LPR 640:1, at 23.5 °C and at 0.5 M urea, reactions were left for >30 min to ensure the folding reaction had reached completion before end-point spectral measurement. Spectra for unfolded OMPs (in 8 M urea) are shown for comparison. Analysis by cold SDS-PAGE demonstrated that OMPs were folded to >90% (MipA does not bandshift, see **Supplementary Figure 2**. Folded BtuB is unstable on SDS-PAGE gels).

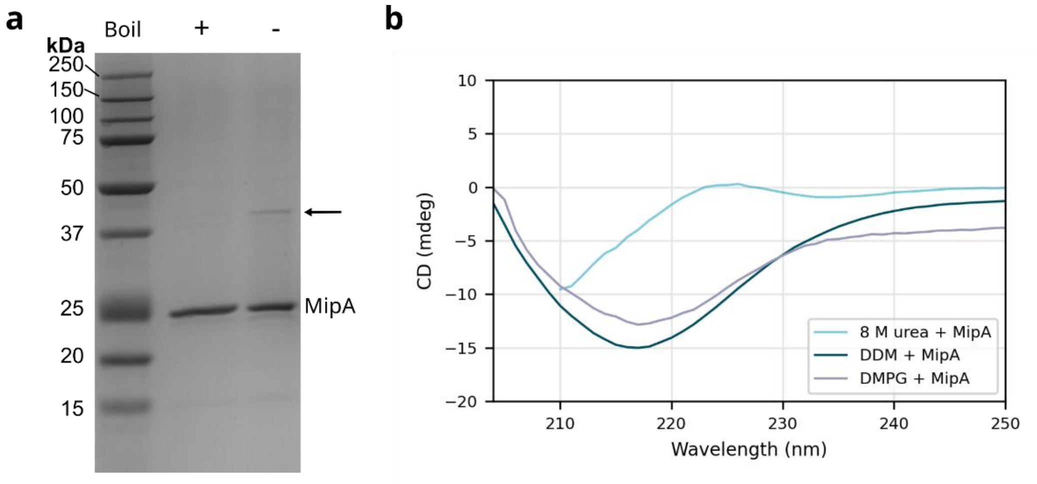

Supplementary Figure 2: MipA characterisation. (a) MipA does not show a folded unfolded bandshift by cold SDS-PAGE, although a minor additional band appears in the unboiled sample (arrow) at approximately the dimer molecular weight (supported by a high confidence dimeric Alphafold structure prediction, **Fig. 6h**). **(b)** Far UV CD indicates formation of β-sheet structure when MipA is refolded into either detergent or DMPG liposomes compared to the unfolded protein in 8 M urea.

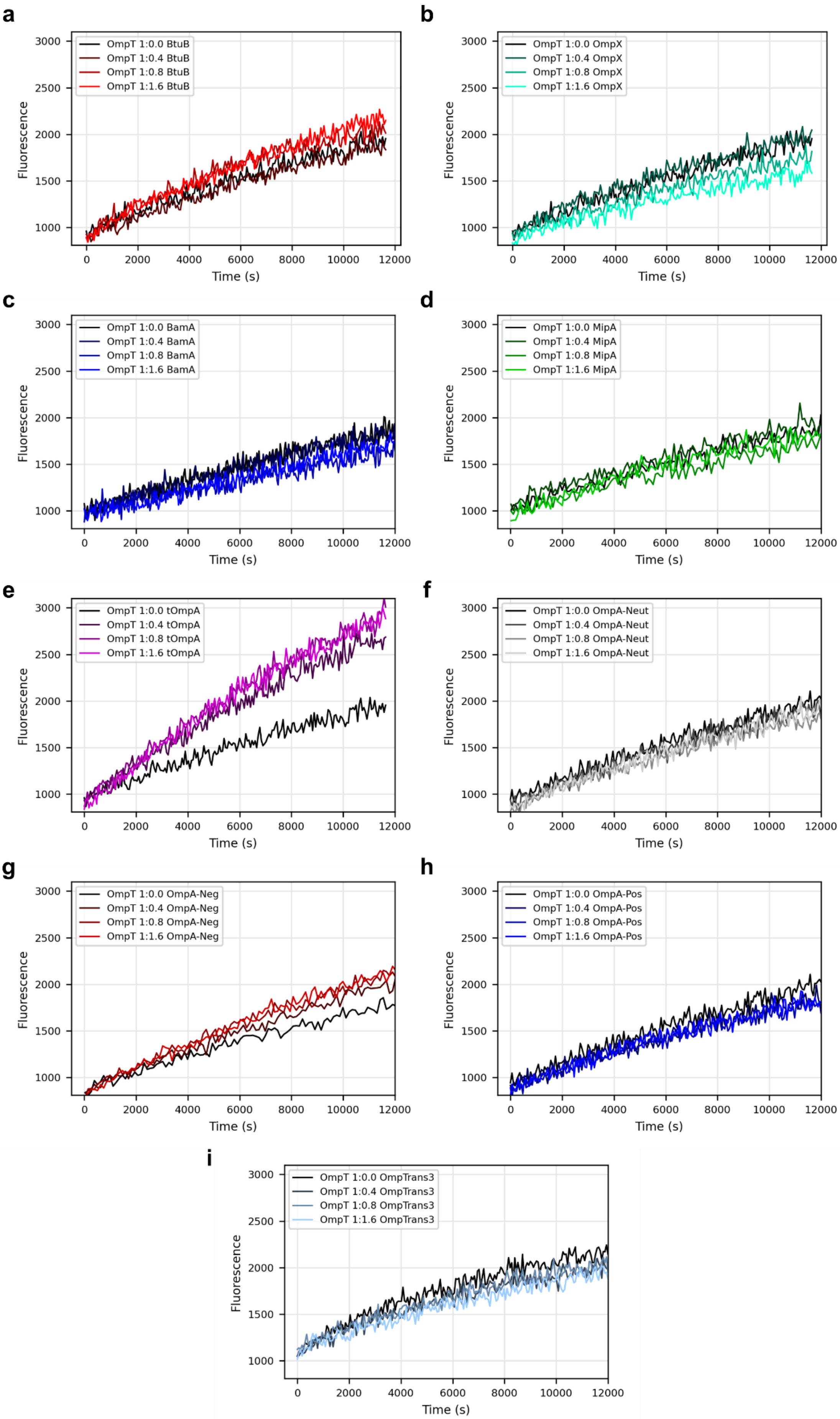

a
OmpT 1:0.0 BtuB
OmpT 1:0.4 BtuB
OmpT 1:0.8 BtuB
OmpT 1:1.6 BtuB
b
OmpT 1:0.0 OmpX
OmpT 1:0.4 OmpX
OmpT 1:0.8 OmpX
OmpT 1:1.6 OmpX
c
OmpT 1:0.0 BamA
OmpT 1:0.4 BamA
OmpT 1:0.8 BamA
OmpT 1:1.6 BamA
d
OmpT 1:0.0 MipA
OmpT 1:0.4 MipA
OmpT 1:0.8 MipA
OmpT 1:1.6 MipA
e
OmpT 1:0.0 tOmpA
OmpT 1:0.4 tOmpA
OmpT 1:0.8 tOmpA
OmpT 1:1.6 tOmpA
f
OmpT 1:0.0 OmpA-Neut
OmpT 1:0.4 OmpA-Neut
OmpT 1:0.8 OmpA-Neut
OmpT 1:1.6 OmpA-Neut
g
OmpT 1:0.0 OmpA-Neg
OmpT 1:0.4 OmpA-Neg
OmpT 1:0.8 OmpA-Neg
OmpT 1:1.6 OmpA-Neg
h
OmpT 1:0.0 OmpA-Pos
OmpT 1:0.4 OmpA-Pos
OmpT 1:0.8 OmpA-Pos
OmpT 1:1.6 OmpA-Pos
i
OmpT 1:0.0 OmpTrans3
OmpT 1:0.4 OmpTrans3
OmpT 1:0.8 OmpTrans3
OmpT 1:1.6 OmpTrans3
Fluorescence
Time (s)
1000
1500
2000
2500
3000
0
2000
4000
6000
8000
10000
12000

Supplementary Figure 3: OmpT-OMP activity traces at increasing OMP concentrations. OmpT activity traces in the absence or presence of another OMP at 0.4-, 0.8-,or 1.2-times molar excess. Titration with increasing concentrations of **(a)** BtuB, **(b)** OmpX, **(c)** BamA, **(d)** MipA, **(e)** tOmpA, **(f)** OmpA-Neut, **(g)** OmpA-Neg, **(h)** OmpA-Pos, and **(i)** OmpTrans3. The concentration of OmpT in all assays was 0.05 µM, and the other OMP at the indicated molar ratiometric concentration, the OmpT peptide substrate was 50 µM.

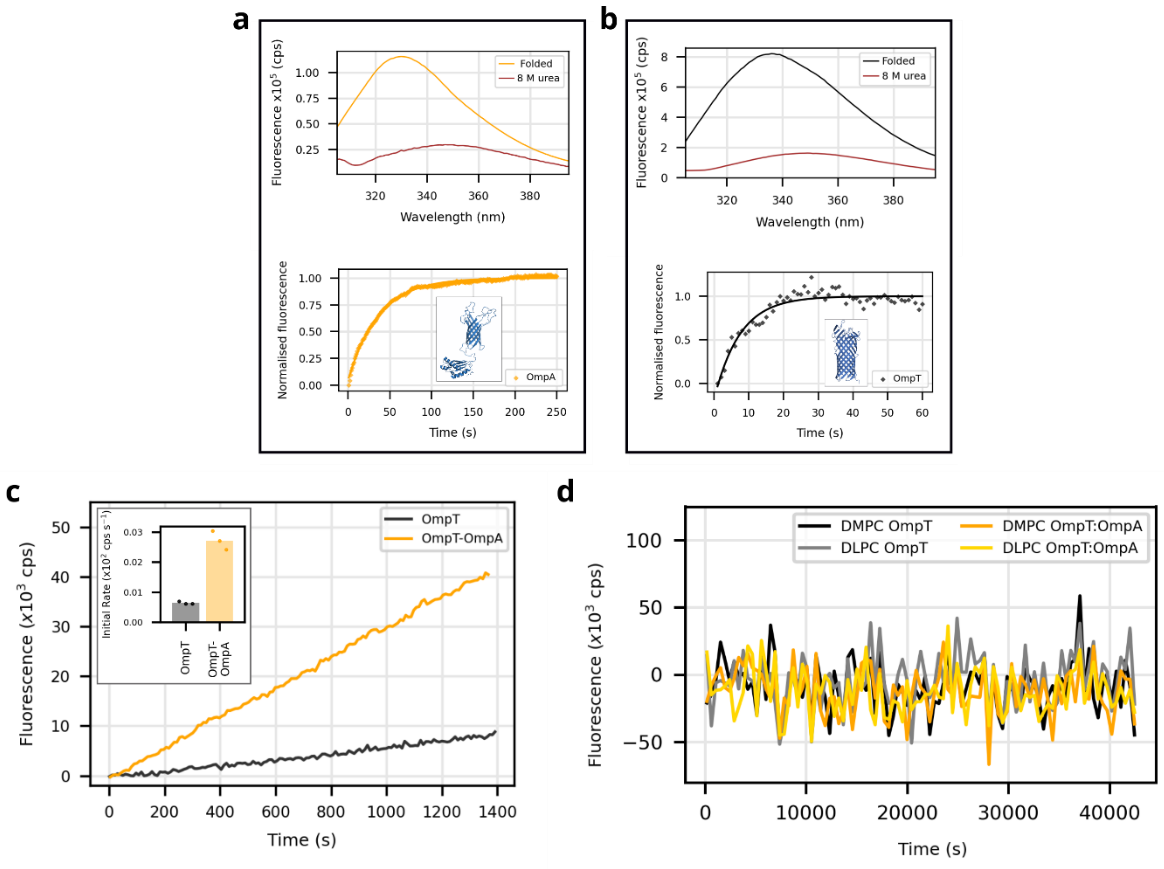

Supplementary Figure 4: OmpT-OmpA activity in DMPS, DLPC and DMPC liposomes. **(a)** OmpA and **(b)** OmpT readily fold into DLPC membranes in 0.5 M urea at 30 °C. For both OMPs the folding kinetics (lower panels, followed by tryptophan fluorescence, fitted to a single exponential (line)) and the folded vs unfolded (8 M urea) intrinsic fluorescence emission spectra (upper plots) are shown. Each protein shows clear, rapid folding kinetics and the expected unfolded-folded spectral transition. **(c)** OmpA enhances OmpT activity in DMPS membranes ~3-fold (n=3). **(d)** OmpT, which is basally inactive in DMPC- and DLPC-membranes, is not activated by OmpA (1:1 molar ratio) (n=4). (In **c-d**, OmpT was 0.05 μM and its peptide substrate was 50 μM).

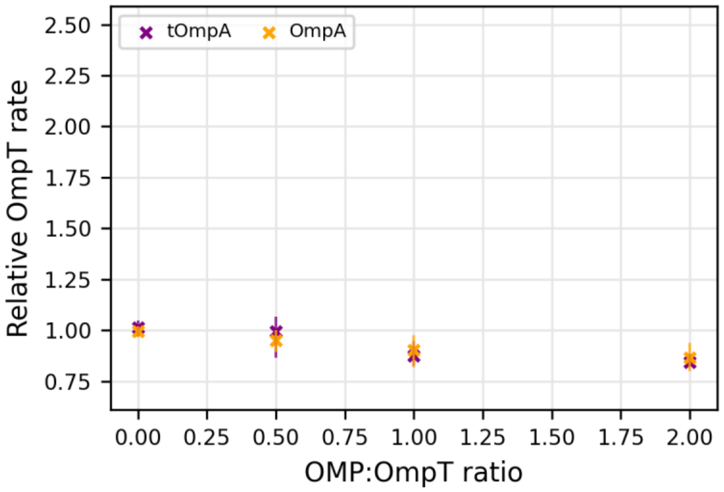

Supplementary Figure 5: OmpA/tOmpA do not activate OmpT when in separate liposomes. Separate proteoliposomes of OmpT and different concentrations of OmpA or tOmpA were prepared and then mixed immediately prior to substrate addition. Empty liposomes were added in the absence of OmpA/tOmpA. (OmpT was 0.05 μM and its peptide substrate was 50 μM).

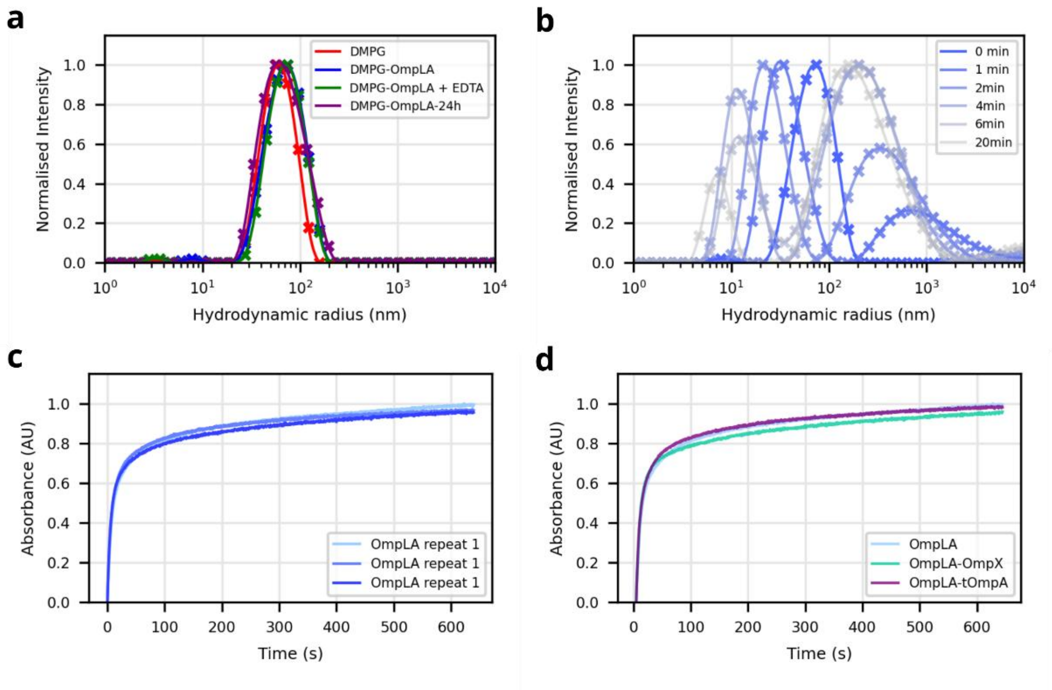

Supplementary Figure 6: OmpLA enzyme activity initiates rapid liposomal collapse. (a) OmpLA refolded into DMPG liposomes is stable for >24 hours in the absence of divalent cations. **(b)** Adding of $CaCl_2$ (1 mM) to activate OmpLA (reaction time-points quenched with excess EDTA) leads to collapse and aggregation of the liposomes from a single ~70 nm peak at 0 min to a dual peak of small fragments and larger aggregates at 20 min. **(c)** Liposome collapse and the formation of large aggregates can be followed by measuring sample optical density (OD) over time (510 nm), yielding reproducible data consistent with the DLS measurements. **(d)** Following OmpLA activity by OD_{510} in the presence of a 1:1 (molar) ratio of OmpX or tOmpA show no significant difference in activity between the samples. (In **b-d**, OmpLA at 0.05 μM).

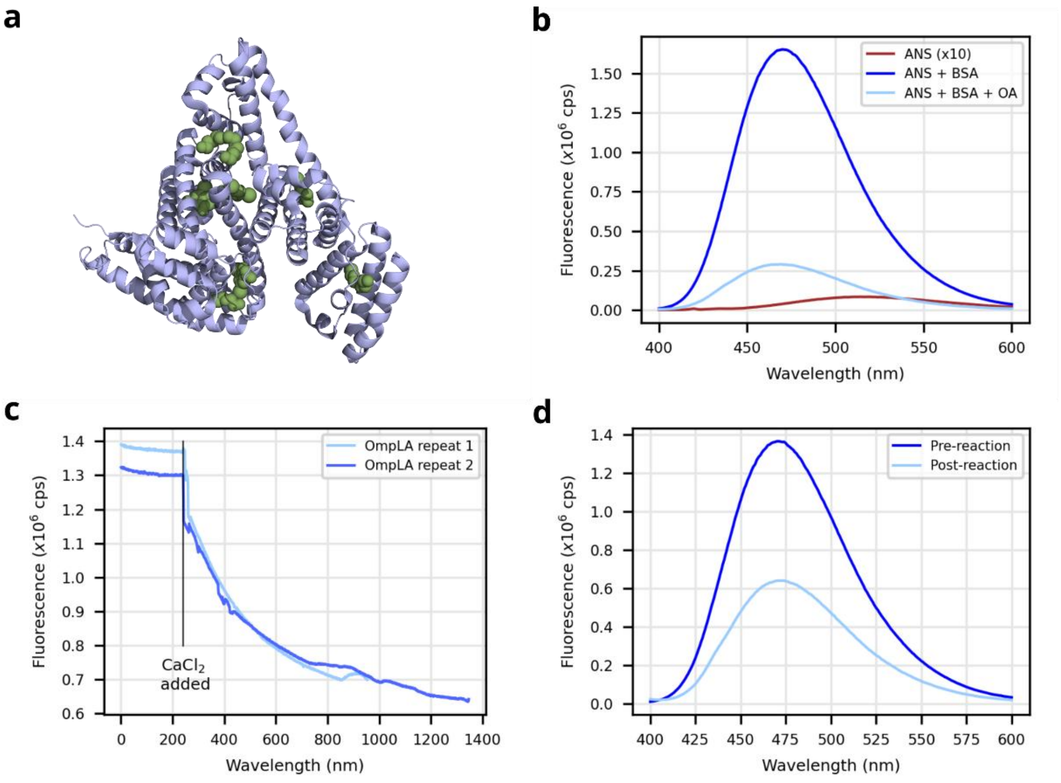

Supplementary Figure 7: OmpLA activity can be measured by detecting free fatty acid (FFA) release with BSA-ANS. (a) Structure of human serine albumin (a homolog of BSA) (blue) bound to six fatty acid molecules bound (green) (PDB: 1BJ5)[46]. **(b)** Increase in ANS fluorescence upon the addition of BSA (dark blue compared to brown), which is reduced upon the addition of oleic acid (OA) (light blue) (20 µM ANS, 1 µM BSA and 0.1% v/v OA). Note also the blue sift in λmax in ANS-BSA compared with ANS alone. **(c)** Monitoring ANS fluorescence over time upon addition of $CaCl_2$ enables the FFA release from DLPS to be monitored (OmpLA at 0.05 µM), and **(d)** fluorescence emission spectra of ANS before and after the reaction show a clear reduction in BSA-ANS fluorescence (shown after 25 min).

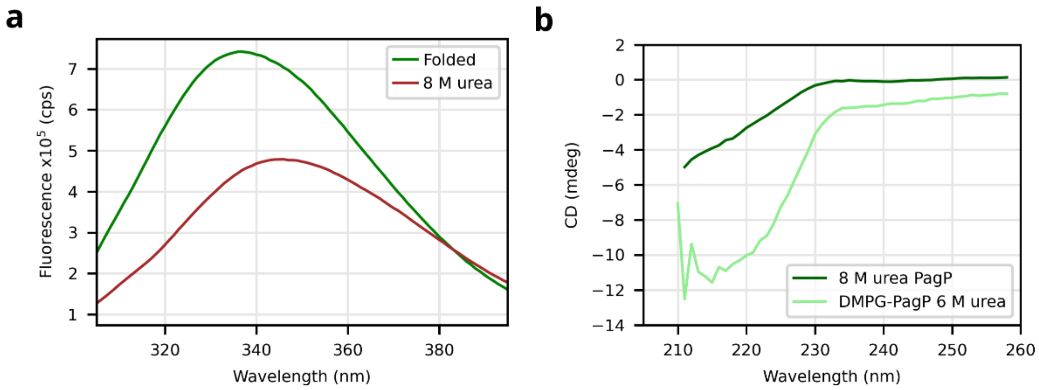

Supplementary Figure 8: PagP folds into DMPG liposomes in the presence of 6 M urea. **(a)** Intrinsic fluorescence and **(b)** background subtracted far UV CD spectra of unfolded (8 M urea) and DMPG-folded PagP (in the presence of 6 M urea).

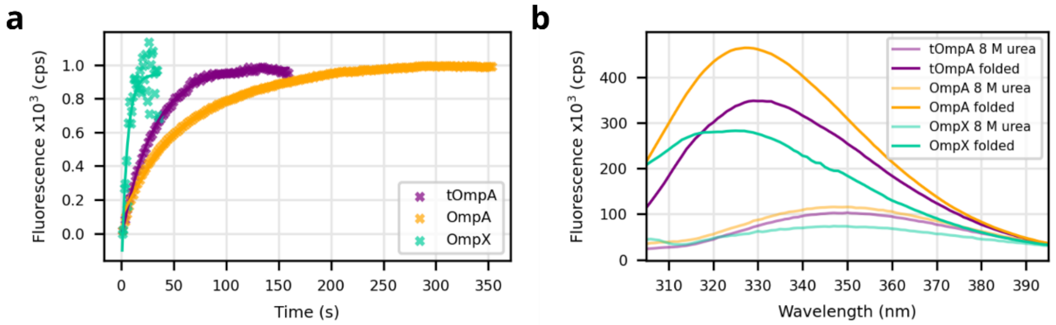

Supplementary Figure 9: OmpA, tOmpA and OmpX fold efficiently into DMPG liposomes in 2 M urea. (a) Folding kinetics monitored using tryptophan fluorescence and **(b)** fluorescence emission spectra of folded and unfolded (8 M urea) proteins, as indicated in the key.

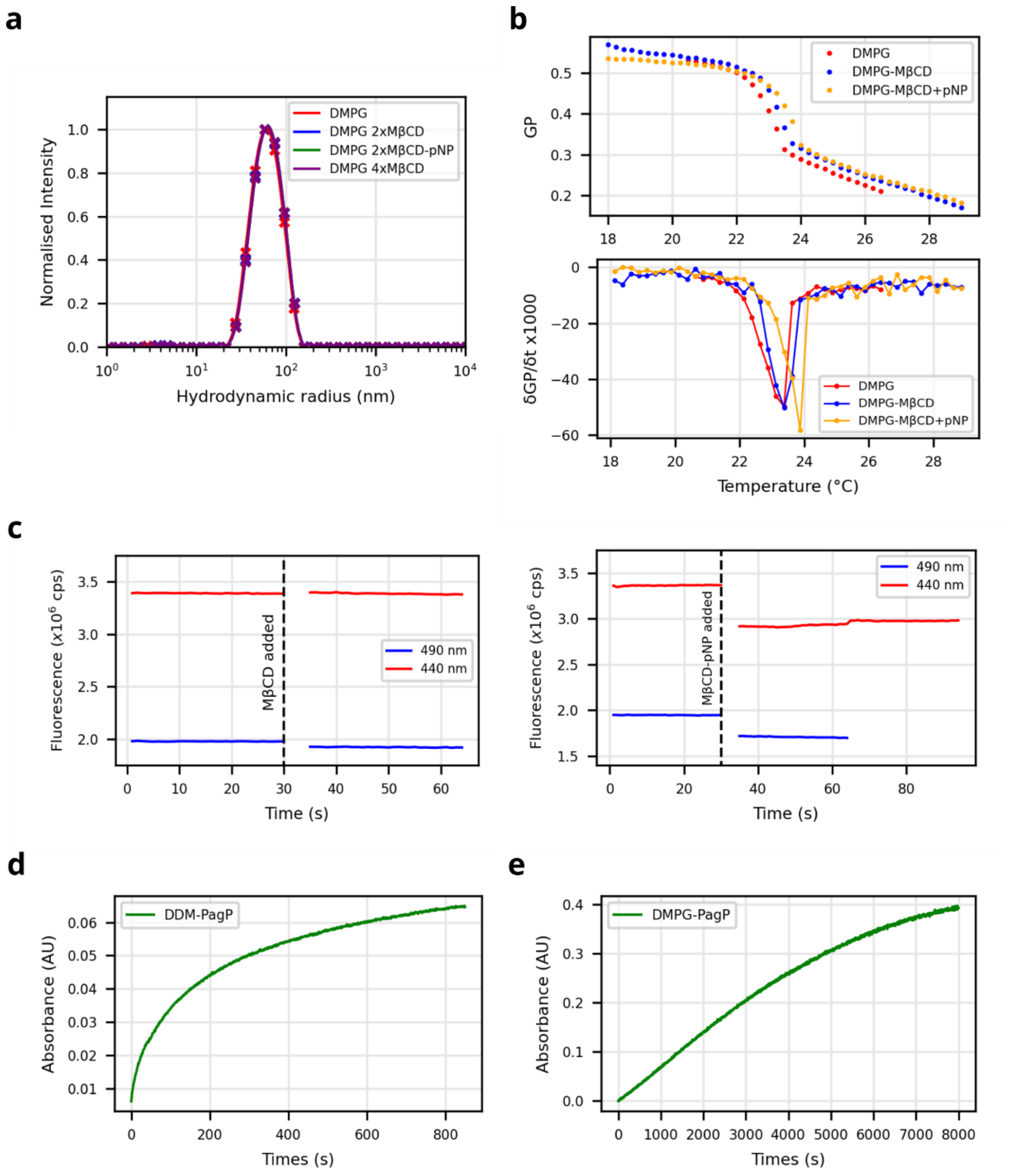

Supplementary Figure 10: PagP activity in liposomes: assay development and validation. (a) DLS shows that liposomes with 1:2 or 1:4 (mol/mol) lipid:MβCD ratios remain intact (up to 2 hours). **(b)** Equilibrium lipid phase transition temperature measured using the laurdan reporter show by the general polarisation (GP, upper) and the first differential of the GP, lower. Transition temperatures for each liposome are determined using the differential peak. **(c)** Time-resolved lipid phase transition temperature measurements upon the addition of MβCD (left) or MβCD-pNP (right), measured at the gel and fluid phase fluorescence peaks of the laurdan reporter (440 nm and 490 nm, respectively). MβCD-pNP can deliver the substrate pNP to **(d)** DDM refolded PagP and **(e)** DMPG refolded PagP to initiate enzyme activity (duplicated from **Fig. 6b** for ease of comparison).

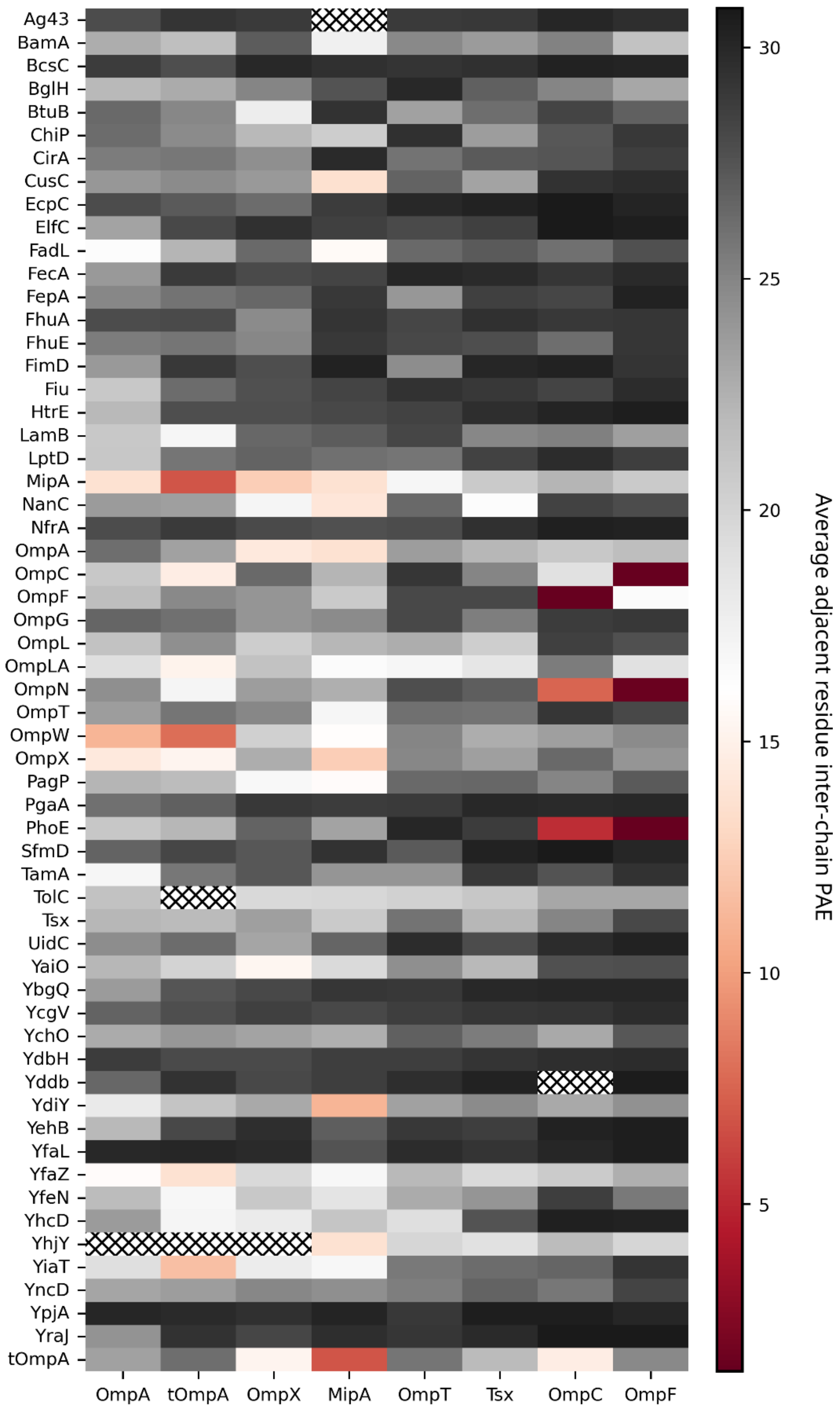

Supplementary Figure 11: Alphafold2 predicted confidence of interactions between seven abundant OMPs and all other known *E. coli* OMPs. Confidence metric is the average (between five predictions) adjacent residue inter-chain PAE (i.e. only considering the PAE between residues of different chains that are geometrically adjacent (1.5 nm cutoff). Hashed data-points indicate the average pLLDT of the model was less than 80. High interchain PAE values indicate low probabilities of interaction, lower values (red) are more likely to interact.

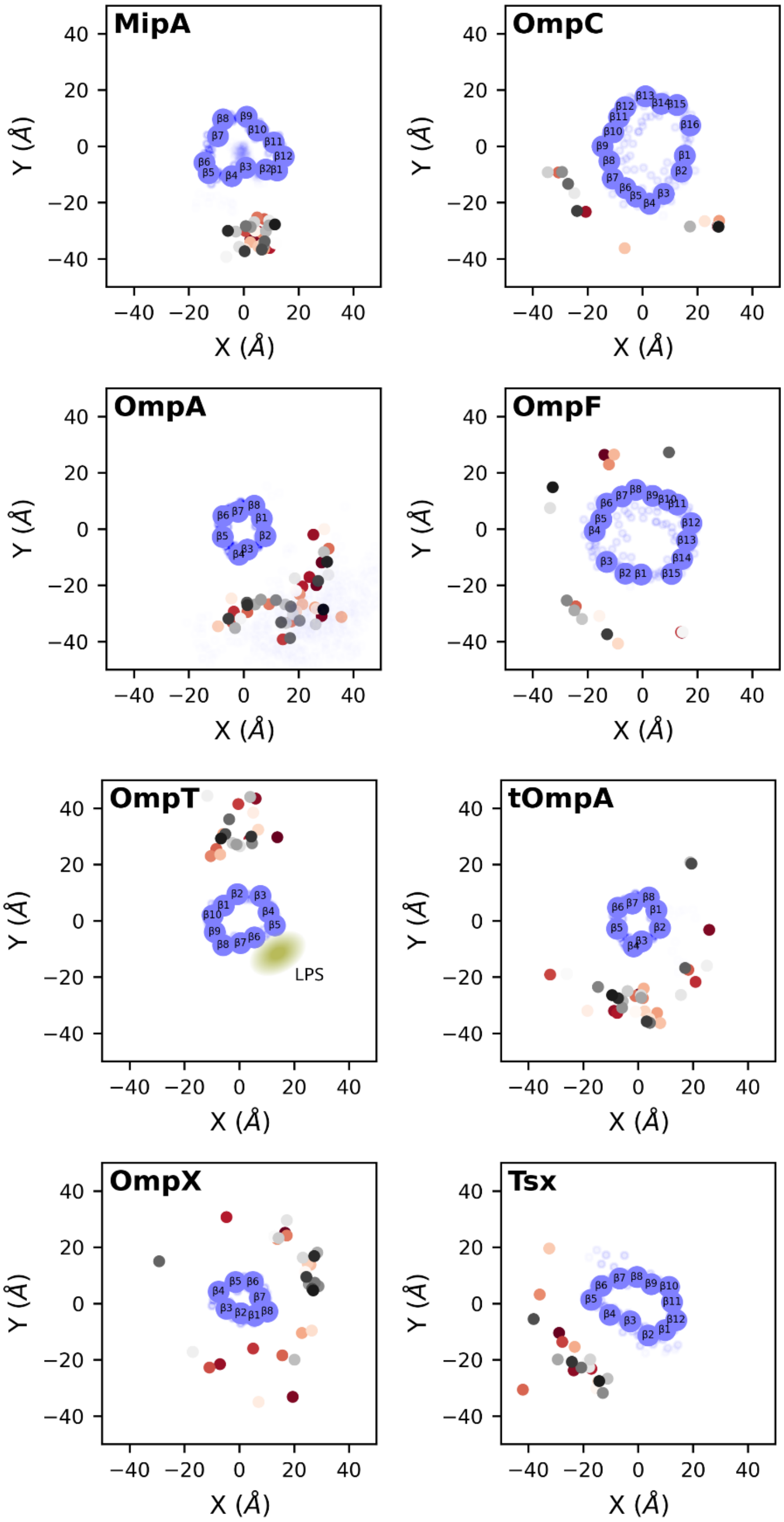

Supplementary Figure 12: Angular distributions of predicted interacting OMPs. All predictions for the abundant OMPs (blue, inter-chain PAE 80) were aligned to the common OMP and the centre of mass of the second OMPs transmembrane domains plotted (dots, black to red based on their inter-chain PAE, as in **Fig. S10**). Models with highly diverging predictions were filtered out based on inter-model RMSD. The green coloured region in the OmpT plot is the expected LPS binding site. See also **Fig. 6b-d**.

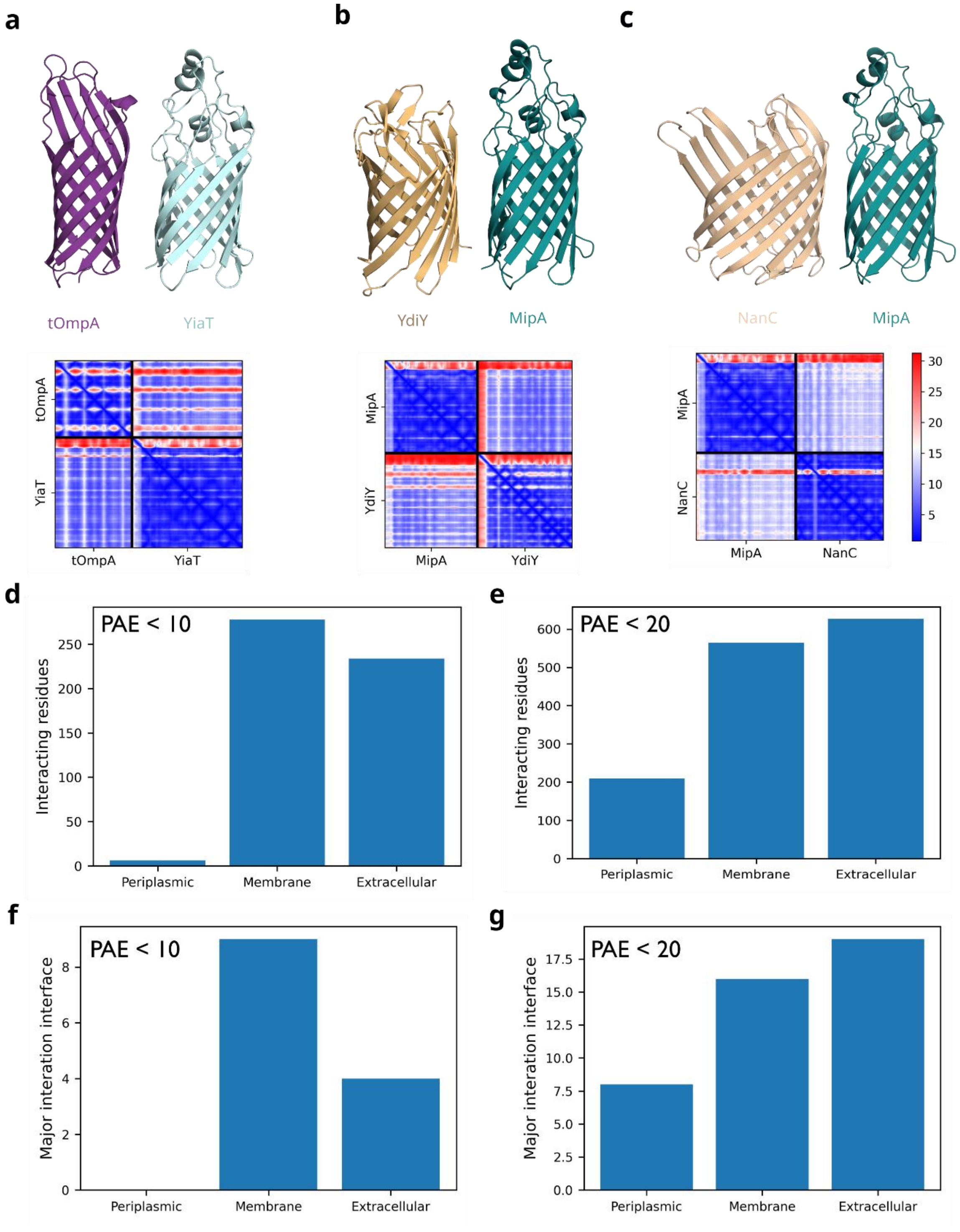

Supplementary Figure 13: Additional high confidence predicted interactions and their PAEs. (a-c) tOmpA-YiaT, YdiT-MipA and NanC-MipA, respectively. For PAE plots, blue indicates high and red low confidence. See also **Fig. 6e-j**. (Interactions between trimeric porin proteins are not shown). Structural location of interacting residues in predictions with inter-chain PAE of **(d)** <10 and **(e)** <20, membrane width determined via the database Orientation of Proteins in the Membrane *Immers* server. Primary (largest) interaction interface for each model with inter-chain PAE of **(f)** <10 and **(g)** <20.

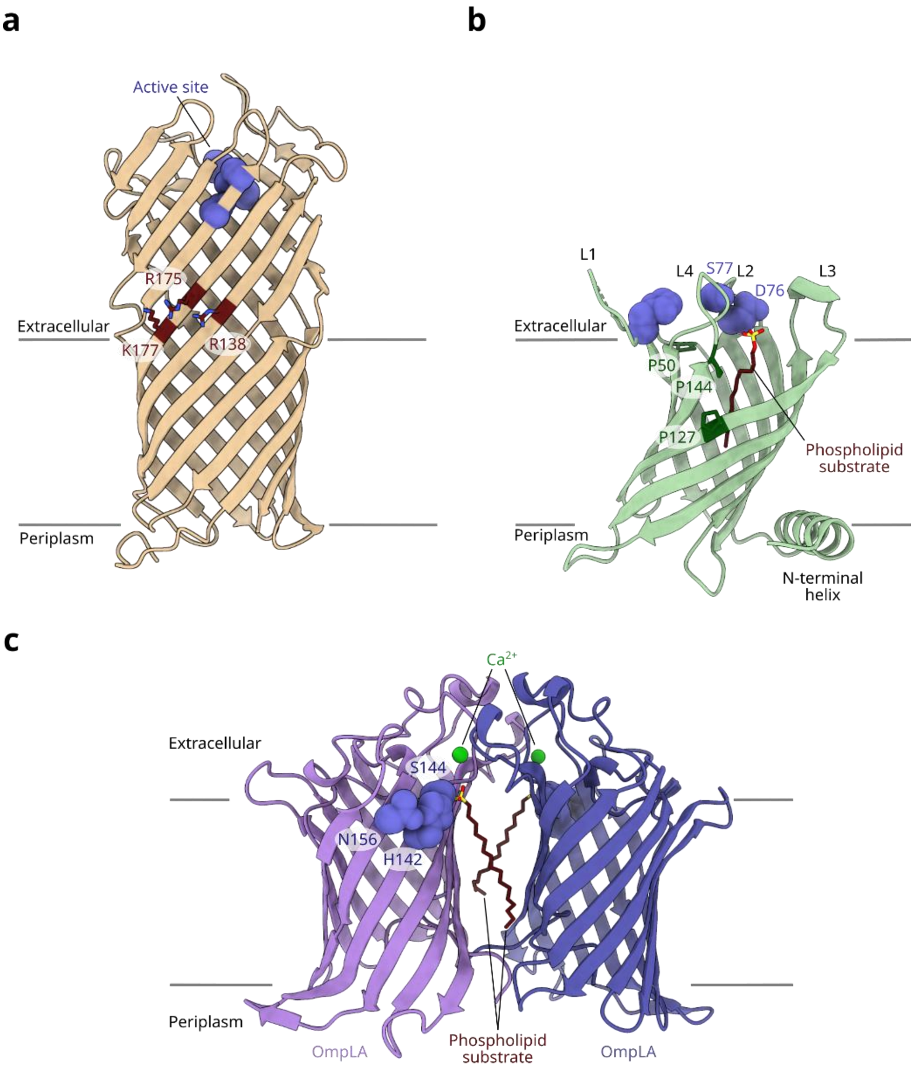

Supplementary Figure 14: Detailed structural comparison of OmpT, PagP and OmpLA. **(a)** OmpT has large, mostly ordered extracellular loops, whose upper cleft contains the active site. Three positive residues (R138, R175, R178) are proposed to form the LPS binding site, interacting with phosphorylations on the Lipid A moiety. **(b)** PagP has minimal extracellular loops and sits in the bilayer at a high tilt, partially anchored by it's N-terminal helix. Substrate access is facilitated by a lateral gate in the OM's outer leaflet between P127 and P144. **(c)** OmpLA strictly requires divalent cations for activity as this mediates the necessary dimerization, with the active sites formed between the two monomers. OmpLA has large extracellular loops at the dimerization interface and much smaller loops away from it. (PDBs: PagP: 3GP6, OmpT: 1I78, OmpLA: 1QD5).
