# Supplementary material for: OmpA Specifically Modulates the Activity of Enzymes that Reside in the Crowded Bacterial Outer Membrane
Source: J Mol Biol. Author manuscript; Available in PMC 2025 Jul 30. (PMC7617971; doi:10.1016/j.jmb.2025.169346)
Supplement: Appendix [file EMS207352-supplement-Appendix.pdf]

Appendix A. Supplementary material

Supplementary material to this article can be found online at https://doi.org/10.1016/j.jmb.2025.169346.
